# Polydopamine‐Modified 2D Iron (II) Immobilized MnPS_3_ Nanosheets for Multimodal Imaging‐Guided Cancer Synergistic Photothermal‐Chemodynamic Therapy

**DOI:** 10.1002/advs.202306494

**Published:** 2023-12-11

**Authors:** Hanhan Xie, Ming Yang, Xiaoli He, Zhen Zhan, Huaide Jiang, Yanmei Ma, Chengzhi Hu

**Affiliations:** ^1^ Shenzhen Key Laboratory of Biomimetic Robotics and Intelligent Systems Department of Mechanical and Energy Engineering Southern University of Science and Technology Shenzhen 518055 China; ^2^ Guangdong Provincial Key Laboratory of Human‐Augmentation and Rehabilitation Robotics in Universities Southern University of Science and Technology Shenzhen 518055 China

**Keywords:** cancer theranostics, chemodynamic therapy, MnPS_3_ nanosheets, photothermal therapy, 2D nanomaterials

## Abstract

Manganese phosphosulphide (MnPS_3_), a newly emerged and promising member of the 2D metal phosphorus trichalcogenides (MPX_3_) family, has aroused abundant interest due to its unique physicochemical properties and applications in energy storage and conversion. However, its potential in the field of biomedicine, particularly as a nanotherapeutic platform for cancer therapy, has remained largely unexplored. Herein, a 2D “all‐in‐one” theranostic nanoplatform based on MnPS_3_ is designed and applied for imaging‐guided synergistic photothermal‐chemodynamic therapy. (Iron) Fe (II) ions are immobilized on the surface of MnPS_3_ nanosheets to facilitate effective chemodynamic therapy (CDT). Upon surface modification with polydopamine (PDA) and polyethylene glycol (PEG), the obtained Fe‐MnPS_3_/PDA‐PEG nanosheets exhibit exceptional photothermal conversion efficiency (*η* = 40.7%) and proficient pH/NIR‐responsive Fenton catalytic activity, enabling efficient photothermal therapy (PTT) and CDT. Importantly, such nanoplatform can also serve as an efficient theranostic agent for multimodal imaging, facilitating real‐time monitoring and guidance of the therapeutic process. After fulfilling the therapeutic functions, the Fe‐MnPS_3_/PDA‐PEG nanosheets can be efficiently excreted from the body, alleviating the concerns of long‐term retention and potential toxicity. This work presents an effective, precise, and safe 2D “all‐in‐one” theranostic nanoplatform based on MnPS_3_ for high‐efficiency tumor‐specific theranostics.

## Graphical abstract

A 2D “all‐in‐one” theranostic nanoplatform based on MnPS_3_ is designed. This nanoplatform shows high photothermal conversion efficiency of 40.7% and excellent pH/NIR‐responsive Fenton catalytic, enabling efficient imaging‐guided synergistic photothermal‐chemodynamic therapy. After fulfilling the therapeutic functions, this nanoplatform can be efficiently excreted from the body to avoid the concerns of long‐term retention and potential toxicity.

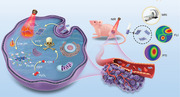

## 1 Introduction

2D nanomaterials have captured tremendous research interests in applications such as electronics, catalysis, energy conversion and storage, as well as biomedicine.^[^ ^1^ ^]^ Nowadays, manganese phosphosulphide (MnPS_3_), a newly emerged and promising member of the 2D metal phosphorus trichalcogenides (MPX_3_) family, has sparked enormous attention due to their diverse chemical compositions and intricate band structures.^[^ ^2^ ^]^ MnPS_3_ maintains its inherent magnetic properties even when reduced to a few layers, possessing a bandgap of ≈3 eV, rendering it a promising candidate for multifunctional electronics/spintronics and battery applications.^[^ ^3^ ^]^ Recent studies have demonstrated that MnPS_3_ nanosheets can be readily obtained through liquid exfoliation procedures due to their inherent layered structures.^[^ ^4^ ^]^ While extensive research has delved into their inherent physicochemical properties and applications in energy storage and conversion,^[^ ^5^ ^]^ their potential in biomedical applications has received relatively limited investigation. MnPS_3_ possesses several unique potential advantages for biomedical applications. Mn is widely utilized in cancer theranostics, and Mn‐based agents have been demonstrated as more promising alternatives for *T*_1_/*T*_2_‐weighted MRI compared to traditional Gd‐based contrast agents.^[^ ^6^ ^]^ Moreover, P and S are considered environmentally friendly elements, and nanomaterials based on them have been embraced as cancer therapeutic agents due to their good biocompatibility and significant promise in biomedical applications.^[^ ^7^ ^]^ Meanwhile, MnPS_3_ possesses a large specific surface area and demonstrates exceptional adsorption capacity for various metal ions such as Ti, Mn, Fe, Co, Cu, Pt, etc.^[^ ^8^ ^]^ These distinctive advantages promote MnPS_3_ as a promising candidate in the field of nanomedicine, particularly for cancer therapy.

Chemodynamic therapy (CDT) is an emerging and alternative tumor therapeutic modality with high tumor specificity and depth independence. CDT leverages the Fenton or Fenton‐like reactions to selectively destroy cancer cells by catalyzing endogenous H_2_O_2_ to produce highly toxic hydroxyl radicals (^•^OH).^[^ ^9^ ^]^ Many transition metal ions, including Fe, Mn, Co, Pt, and Cu, can act as Fenton or Fenton‐like agents within the tumor microenvironment (TME) to facilitate the conversion of the endogenous H_2_O_2_ into ^•^OH.^[^ ^10^ ^]^ Among them, Fe (II) is regarded as the most efficient Fenton agent for CDT.^[^ ^11^ ^]^ Unfortunately, dissociative Fe ions can hardly passively accumulate within malignant tissues through the enhanced permeability and retention (EPR) effect.^[^ ^12^ ^]^ When Fe (II) is administered directly and non‐specifically as a CDT agent, it can lead to oxidative stress throughout the entire body.^[^ ^13^ ^]^ Additionally, Fe (II) is prone to oxidation to Fe (III), resulting in precipitation and cessation of its involvement in the Fenton reaction.^[^ ^14^ ^]^ Hence, the development of an intelligent Fe (II) delivery system is crucial to effectively prevent oxidation and leakage during circulation, while simultaneously achieving controlled release at specific locations.

In addition to CDT, photothermal therapy (PTT) exhibits an elevated tumor eradication efficiency and minimal adverse effects, which has received considerable attention in cancer therapy. However, due to the limited depth of light penetration and inhomogeneous heat distribution during irradiation, the growth of tumors is difficult to inhibit completely by a single PTT. It has been reported that the hyperthermia induced by PTT not only induces cancer cell death but also enhances the production of ^•^OH in CDT, leading to a synergistic effect.^[^ ^9^, ^15^ ^]^ Consequently, the rational combination of CDT and PTT is anticipated to significantly elevate the synergistic therapeutic efficacy compared to the single therapeutic modality. Moreover, insufficient visualization during the therapeutic process significantly hinders the application of CDT and PTT. To address these issues, the development of a multimodal nanoplatform capable of achieving effective CDT and PTT while enabling multimodal imaging becomes crucial for precise and efficient cancer therapy.

**Figure 1:**
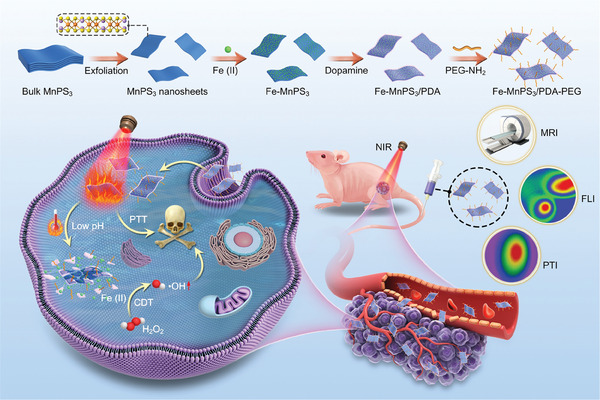
Schematic illustration of the procedure for synthesizing Fe‐MnPS_3_/PDA‐PEG nanosheets and their applications in bioimaging‐guided synergistic photothermal‐chemodynamic therapy for cancer.

Herein, for the first time, we present the design and fabrication of a 2D “all‐in‐one” theranostic nanoplatform based on MnPS_3_ nanosheets for multimodal imaging‐guided synergistic PTT/CDT (**Figure** **1**). By utilizing the ultra‐high specific surface area and exceptional adsorption capacity for metal ions of MnPS_3_ nanosheets, we immobilize Fe (II) on the surface of MnPS_3_ nanosheets via electrostatic absorption. MnPS_3_ nanosheets can immobilize Fe (II) with high efficacy and protect Fe (II) from oxidation and aggregation. The immobilization of Fe (II) not only enhances the photothermal performance of the MnPS_3_ nanosheets but also facilitates effective CDT. Additionally, to improve the photothermal performance of the MnPS_3_‐based co‐delivery platform and prevent the leakage of Fe (II), while simultaneously achieving controlled release at specific locations, PDA was employed for surface modification of the Fe‐MnPS_3_ nanosheets via the dopamine polymerization method. After PDA modification, the photothermal capabilities and pH/NIR‐responsive Fe ions release activity can be significantly enhanced. More importantly, this designed platform can facilitate multimodal imaging‐guided synergistic photothermal‐chemodynamic therapy and be efficiently excreted from the body after fulfilling the therapeutic functions. Our work constitutes a comprehensive, closed‐loop approach, with highly efficient PTT and 2D Fenton agents as well as multimodal imaging simultaneously achieved in a single 2D platform, leading to efficient tumor eradication. And this nanoplatform can be efficiently excreted from the body, a feature often overlooked in other research papers. We anticipate this work will provide an effective, precise, and safe 2D “all‐in‐one” theranostic nanoplatform for high‐efficiency tumor‐specific theranostics.

## 2 Results and Discussion

### 2.1 Synthesis and Characterization of MnPS_3_ Nanosheets

**Figure 2:**
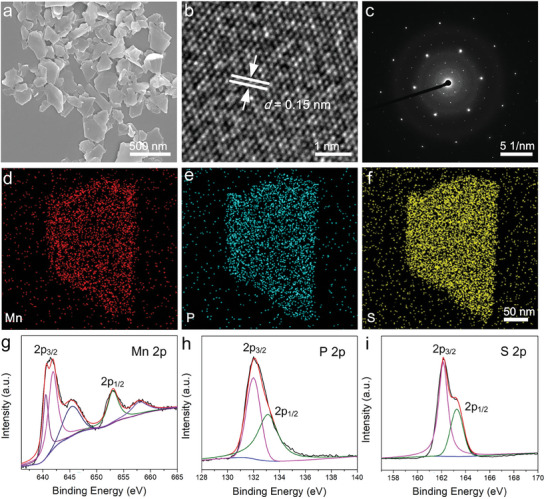
Characterization of MnPS_3_ nanosheets: a) SEM image. b) HR‐TEM image. c) SAED pattern. d–f) EDS mapping. g–i) XPS spectra of g) Mn 2p, h) P 2p, and i) S 2p.

The MnPS_3_ nanosheets were prepared by using a straightforward liquid exfoliation technique, as previously reported.^[^ ^16^ ^]^ Briefly, the bulk MnPS_3_ crystals were ground into fine powders and then subjected to ultrasound probe sonication in N‐methyl‐2‐pyrrolidone (NMP). Powder X‐ray diffraction (XRD) was carried out to examine the crystal phase and purity of the synthesized MnPS_3_ nanosheets. As illustrated in Figure S1 (Supporting Information), the XRD pattern of the MnPS_3_ nanosheets is in accordance with the bulk MnPS_3_ crystal and matches JCPDS card No. 33–0903, confirming the preservation of the crystal structure during the exfoliation process. The morphology of the MnPS_3_ nanosheets was observed by scanning electron microscopy (SEM), as shown in **Figure** **2**a. The MnPS_3_ nanosheets have a relatively uniform 2D sheet‐like morphology with an average lateral size of 220 nm based on the statistical results from the SEM images (Figure S2, Supporting Information). The high‐resolution transmission electron microscopy (HR‐TEM) in Figure 2b reveals its excellent crystallinity, with lattice fringes distance of 0.15 nm, indexing to the (001) plane of MnPS_3_.^[^ ^17^ ^]^ The selected area electron diffraction (SAED) pattern (Figure 2c) confirms the high‐quality monocrystalline nature of the MnPS_3_ nanosheets. Figure S3a (Supporting Information) shows the atomic force microscopy (AFM) images and the corresponding height profile of the representative MnPS_3_ nanosheets with thicknesses of 4.6–5.8 nm (Figure S3b, Supporting Information), implying the MnPS_3_ nanosheets structure with only a few layers. The elemental composition of the MnPS_3_ nanosheets was analyzed using energy‐dispersive X‐ray spectroscopy (EDS). The EDS mapping depicted in Figure 2d–f discloses the uniform spatial distribution of the Mn, P, and S elements throughout the nanosheets. To analyze the surface chemical states of the MnPS_3_ nanosheets, X‐ray photoelectron spectroscopy (XPS) was further conducted. Figure 2g presents the high‐resolution XPS spectrum of Mn, in which the strong peaks located at 640.5 and 653.0 eV are identified as 2p_3/2_ and 2p_1/2_ of Mn^2+^. The satellite peaks at 645.3 and 658.0 eV arising from the coupling of 3p‐3d at the optical absorption edge, while the P 2p core level spectrum in Figure 2h shows strong peaks at binding energies of 132.0 and 133.1 eV assigned to 2p_3/2_ and 2p_1/2_ levels, respectively. Similarly, in the S 2p core level spectrum in Figure 2i, two characteristic peaks appear at 162.1 and 163.2 eV, corresponding to 2p_3/2_ and 2p_1/2_ levels, respectively.^[^ ^18^ ^]^ Furthermore, the Raman scattering spectrum in Figure S4 (Supporting Information) displays three pronounced peaks at 150, 275, and 395 cm^−1^, which are correlated to the A_1g_ and E_2g_ modes of MnPS_3_ nanosheets, respectively.^[^ ^19^ ^]^ These results demonstrate the successful synthesis of the 2D MnPS_3_ nanosheets.

### 2.2 Synthesis and Characterization of Fe‐MnPS_3_/PDA‐PEG Nanosheets

**Figure 3:**
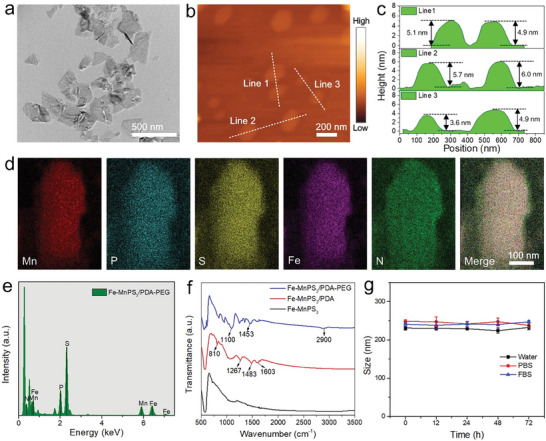
Characterization of MnPS_3_‐based nanomaterials: a) TEM image and b) AFM image of Fe‐MnPS_3_ nanosheets. c) Height profiles of Fe‐MnPS_3_ nanosheets along the white lines in b). d) EDS mapping of Mn, P, S, Fe, N, and merge for Fe‐MnPS_3_/PDA‐PEG nanosheets, respectively. e) EDS spectrum of Fe‐MnPS_3_/PDA‐PEG nanosheets. f) FTIR spectra of Fe‐MnPS_3_, Fe‐MnPS_3_/PDA, and Fe‐MnPS_3_/PDA‐PEG nanosheets. g) Size changes of Fe‐MnPS_3_/PDA‐PEG nanosheets in different physiological media, including water, PBS, and FBS, at different time points.

The immobilization of Fe (II) ions on the 2D MnPS_3_ nanosheets (Fe‐MnPS_3_) was carried out following a previously described method.^[^ ^14^ ^]^ As characterized by TEM in **Figure** **3**a, the Fe‐MnPS_3_ nanosheets maintain a sheet‐like morphology similar to that of the bare MnPS_3_ nanosheets, which indicates that the Fe (II) ions modification does not significantly change the morphology of MnPS_3_ nanosheets. The thickness of the Fe‐MnPS_3_ nanosheets was determined by AFM, as shown in Figure 3b,c, with typical nanosheets exhibiting thicknesses in the range of 3.6–6.0 nm. Furthermore, EDS mapping in Figure S5 (Supporting Information) reveals that the Fe elements are homogeneously dispersed in Fe‐MnPS_3_ nanosheets, with a strong signal that closely overlaps with Mn, P, and S elements of MnPS_3_ nanosheets. The EDS mapping result indicates that the Fe ions are successfully immobilized on the MnPS_3_ nanosheets. The XRD pattern of Fe‐MnPS_3_ in Figure S6 (Supporting Information) does not display any observed specific peaks originating from the lattice plane of Fe, suggesting that Fe ions are adsorbed onto the surface of MnPS_3_ nanosheets rather than forming crystal structures.^[^ ^14^ ^]^ The chemical composition and states of Fe‐MnPS_3_ nanosheets were investigated by X‐ray photoelectron spectroscopy (XPS). As shown in Figure S7 (Supporting Information), compared with the pure MnPS_3_ nanosheets, the high‐resolution XPS spectra of Mn, P, and S show no obvious peak change after Fe ions immobilization. To further investigate the chemical state of the immobilized Fe ions on the MnPS_3_ nanosheets, a high‐resolution Fe 2p XPS spectrum was further conducted. As shown in Figure S8 (Supporting Information), two main peaks located at 709.0 and 722.6 eV are assigned to Fe 2p_3/2_ and Fe 2p_1/2_ of Fe (II),^[^ ^20^ ^]^ while a specific Fe(II) satellite is observed at 729.4 eV attributing to Fe 2p_1/2_.^[^ ^14^ ^]^ According to these peaks, the major chemical state of the adsorbed Fe ions is Fe (II) rather than Fe (III), suggesting that Fe (II) ions are prevented from oxidizing to Fe (III) to a certain extent. Meanwhile, the Fe‐MnPS_3_ nanosheets exhibit enhanced water stability compared to pure MnPS_3_ nanosheets (Figure S9, Supporting Information). To evaluate the Fe (II) ion immobilization ability, MnPS_3_ dispersions with the same volume but various concentrations (0, 20, 40, 100, 200, and 400 ppm) were prepared and mixed into the FeSO_4_^•^7H_2_O solution under magnetic stirring. These FeSO_4_^•^7H_2_O solutions have identical concentration and volume to ensure an equal initial amount of Fe (II) ions. The precipitate containing Fe (II) ions was collected after discarding the supernatant by centrifugation, and Fe element concentrations were detected using inductively coupled plasma mass spectrometry (ICP‐MS). As depicted in Figure S10 (Supporting Information), the efficiency of immobilizing Fe ions increases as the MnPS_3_ nanosheet concentrations rise. These findings confirm that MnPS_3_ nanosheets can efficiently serve as nanoplatforms for Fe (II) ions immobilization.

To improve the photothermal performance of the MnPS_3_‐based co‐delivery platform and prevent the leakage of Fe (II), PDA was employed for surface modification of the Fe‐MnPS_3_ nanosheets through self‐polymerization of DA under weakly alkaline conditions.^[^ ^21^ ^]^ Moreover, to further enhance its stability, biocompatibility, and prolong its in vivo circulation time, PEG‐NH_2_ modification was performed via electrostatic adsorption. The EDS mapping images in Figure 3d show that the illustrative elements, including Mn, P, S, Fe, and N, are evenly distributed on the Fe‐MnPS_3_/PDA‐PEG nanosheets. The characteristic peaks of each element were further assessed by the EDS spectrum (Figure 3e). To detect the contents of Fe and Mn in the Fe‐MnPS_3_/PDA‐PEG nanosheets, we employed the ICP‐MS analysis. As shown in Table S1 (Supporting Information), the contents of Fe and Mn account for ≈30.1% and 30.2% of the total mass of Fe‐MnPS_3_/PDA‐PEG nanosheets, respectively. To verify the successful grafting of PDA and PEG‐NH_2_ onto the nanosheets, we conducted Fourier transform infrared spectroscopy (FTIR) analysis on Fe‐MnPS_3_, Fe‐MnPS_3_‐PDA, and Fe‐MnPS_3_/PDA‐PEG, as shown in Figure 3f. After PDA modification, four new absorption peaks emerge ≈810, 1267, 1483, and 1604 cm^−1^, which can be assigned to C─H out‐of‐plane bending vibration, C‐N stretching vibrations, C─C and C═C stretching vibrations, respectively.^[^ ^22^ ^]^ These findings suggest the successful modification of PDA onto the Fe‐MnPS_3_ nanosheets. Furthermore, after the reaction with PEG‐NH_2_, three distinct characteristic peaks appear at ≈1100, 1455, and 2900 cm^−1^, corresponding to C─O, C─N, and ─CH_2_ stretching vibrations,^[^ ^23^ ^]^ respectively. These results provide further evidence of the covalent bonding of PEG‐NH_2_ chains onto the surface of the nanosheets. In addition, the stepwise changes in the zeta potentials of the various samples range from −9.6 mV to −7.6, −12.3, and −9.4 mV, further confirming the successful loading of Fe (II) ions and the effective modification of PDA and PEG‐NH_2_ (Figure S11, Supporting Information). The size distribution of Fe‐MnPS_3_/PDA‐PEG nanosheets was further determined by dynamic light scattering (DLS). The results presented in Figure S12 (Supporting Information) reveal that the hydrodynamic particle size was 234.0 nm, and the particles exhibit a low value of polydispersity index (PDI) of 0.125, which indicates the particles possess uniform sizes. To evaluate the stability of Fe‐MnPS_3_/PDA‐PEG nanosheets, the long‐term hydrodynamic size changes in different physiological media such as water, phosphate‐buffered saline (PBS), and fetal bovine serum (FBS) were measured. As depicted in Figure 3g, there are no significant changes in size during 72 h, indicating that the prepared nanosheets display excellent stability. Besides, the Fe‐MnPS_3_/PDA‐PEG nanosheets were further dispersed in the above solvents for 72 h. The photographs in Figure S13 (Supporting Information) show their outstanding stability and good dispersion without any macroscopic aggregates. These results suggest that nanosheets can be further employed in biological systems for biomedical applications.

### 2.3 Photothermal and Chemodynamic Performances of Fe‐MnPS_3_/PDA‐PEG Nanosheets

**Figure 4:**
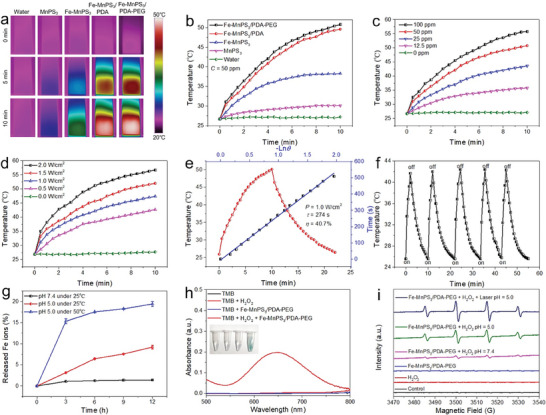
CDT and PTT performances of Fe‐MnPS_3_/PDA‐PEG nanosheets: a) Infrared thermal images of water, MnPS_3_, Fe‐MnPS_3_, Fe‐MnPS_3_/PDA, and Fe‐MnPS_3_/PDA‐PEG with the same MnPS_3_ concentration (50 ppm) in quartz cuvette after irradiation for 0, 5, and 10 min under an 808 nm laser (1.0 W cm^−2^). b) Temperature changes of the above samples. c) PT heating curves of the Fe‐MnPS_3_/PDA‐PEG nanosheets dispersed in water with various concentrations (0, 12.5, 25, 50, and 100 ppm) under irradiation with an 808 nm laser (1.0 W cm^−2^). d) PT heating curves of the Fe‐MnPS_3_/PDA‐PEG nanosheets with different powder densities (0, 0.5, 1.0, 1.5, and 2.0 W cm^−2^). e) Photothermal conversion efficiency of Fe‐MnPS_3_/PDA‐PEG nanosheets. Red line: PT effects of Fe‐MnPS_3_/PDA‐PEG nanosheets under 808 nm laser irradiation for 10 min; Blue line: Time constant (*τ*_s_) measured during the cooling period. f) PT stability of Fe‐MnPS_3_/PDA‐PEG nanosheets after five laser on/off cycles. g) Quantified Fe ions released from Fe‐MnPS_3_/PDA‐PEG nanosheets under different pH and temperature conditions. h) UV–vis absorption spectra of oxTMB under different conditions (only TMB, TMB + H_2_O_2_, TMB + Fe‐MnPS_3_/PDA‐PEG, and TMB + Fe‐MnPS_3_/PDA‐PEG + H_2_O_2_). Inset: corresponding photographs of each group. i) EPR spectra of different reaction systems with the 5,5‐dimethyl‐1‐pyrroline‐N‐oxide (DMPO) as a spin‐trapping agent.

To explore the potential of Fe‐MnPS_3_/PDA‐PEG nanosheets as a PTT agent, the photothermal conversion characteristics were further assessed. After irradiation by an 808 nm laser for 10 min, the resulting infrared thermal images of water, MnPS_3_, Fe‐MnPS_3_, Fe‐MnPS_3_/PDA, and Fe‐MnPS_3_/PDA‐PEG are recorded in **Figure** **4**a. Obviously, after PDA and PEG modification, the nanosheets exhibit a prominent photothermal heating effect. The temperature changes in Figure 4b demonstrates similar results. Notably, the immobilization of Fe (II) ions on MnPS_3_ nanosheets remarkably boosts the photothermal conversion effect compared to pure MnPS_3_ nanosheets, indicating the potential of photothermal synergistic effects between MnPS_3_ and Fe (II) ions. The photothermal heating effect of the Fe‐MnPS_3_/PDA‐PEG nanosheets with various concentrations (0, 12.5, 25, 50, and 100 ppm) was evaluated under an 808 nm laser with a power density of 1.0 W/cm^2^ (Figure 4c). A pronounced concentration‐dependent temperature elevation is observed with increasing concentrations. Particularly, at a concentration of 50 ppm, the temperature increases dramatically to 50.8°C during a 10 min irradiation period, a temperature level sufficient to induce cancer cell death. In comparison, pure water exhibits negligible temperature escalation under identical conditions. Furthermore, we evaluated the photothermal response with varying laser power densities (0, 0.5, 1.0, 1.5, and 2.0 W cm^−2^) (Figure 4d), which confirmed the thermal effect's dependence on power density. Moreover, a vital parameter to evaluate photothermal capability is photothermal conversion efficiency. Based on the calculation formula reported by previous literature,^[^ ^24^ ^]^ the photothermal conversion efficiency (*η*) of the Fe‐MnPS_3_/PDA‐PEG is determined to be 40.7% (Figure 4e), which is notably higher than that of the most commonly reported nanomaterials such as Au nanoshells (13%),^[^ ^25^ ^]^ Au nanorods (21%),^[^ ^26^ ^]^ Cu_9_S_5_ nanoflowers (25.7%),^[^ ^27^ ^]^ black phosphorus QDs (28.4%),^[^ ^28^ ^]^ Bi_2_Se_3_ nanosheets (34.6%),^[^ ^29^ ^]^ and Ti_3_C_2_ nanosheets (30.6%).^[^ ^30^ ^]^ To assess the photothermal stability of Fe‐MnPS_3_/PDA‐PEG nanosheets, five lasers on/off repetitive cycles are used. As shown in Figure 4f, no significant deterioration is observed during temperature elevation. Besides, the particle size and absorption of the nanosheets before and after NIR irradiation were further analyzed. The results confirm that NIR irradiation has no obvious effect on size and absorption properties, corroborating the excellent photothermal stability of Fe‐MnPS_3_/PDA‐PEG nanosheets (Figure S14, Supporting Information).

The pH‐responsive Fe ions release performance was further performed. The buffer solutions at pH 5.0 and 7.4 were used to simulate the tumor microenvironment and normal tissue, respectively. As depicted in Figure 4g, the release of Fe ions is pH‐dependent and can be greatly enhanced in acidic environments. The pH‐dependent behavior can be attributed to the pH sensitivity of PDA.^[^ ^31^ ^]^ In a neutral environment, the PDA formed on the surface of the nanosheets acts as a protected layer to effectively suppress the rapid release of Fe ions. However, in acidic conditions, the external PDA coating gradually degrades and peels off from the nanosheets, leading to accelerated Fe ions release. Since the in vivo tumor microenvironment and the interior of endosomes/lysosomes within cells are typically acidic (pH = 4.5–6.0), while the bloodstream is neutral,^[^ ^32^ ^]^ the preferential release of Fe ions in acidic environments can minimize the risks to normal cells, thus reducing potential bodily toxicity. Moreover, when subjected to heating at 50 °C under pH 5.0, the release of Fe ions significantly increased. This can be attributed to the combination of the acidic environment and elevated temperature, which accelerates the degradation of PDA and results in the rapid release of Fe ions. Meanwhile, the acidic environment as well as temperature elevation can also promote the rapid release of Mn ions under the same conditions (Figure S15, Supporting Information).

To validate the Fenton catalytic activity, the colorless substrates 3,3′,5,5′‐tetramethylbenzidine (TMB) were further adopted as an indicator. TMB can be oxidized by ^•^OH to produce blue oxTMB, which exhibits strong absorption in the spectra range of 500–750 nm.^[^ ^33^ ^]^ After incubating Fe‐MnPS_3_/PDA‐PEG nanosheets with H_2_O_2_ and TMB, the initially colorless TMB gradually turned blue with an obvious absorption increase from 500 to 750 nm, indicating the catalytic generation of ^•^OH in solution (Figure 4h). These results are further confirmed by a typical colorimetric assay based on the degradation of methylene blue (MB), where a visible decrease in absorbance is observed when MB is incubated with Fe‐MnPS_3_/PDA‐PEG nanosheets and H_2_O_2_ (Figure S16, Supporting Information). In contrast, no significant variation in MB absorbance in the absence of H_2_O_2_ or Fe‐MnPS_3_/PDA‐PEG nanosheets, providing clear evidence for the occurrence of the Fenton reaction. Additionally, to more intuitively demonstrate that the Fe‐MnPS_3_/PDA‐PEG nanosheets can generate ^•^OH, the electron paramagnetic resonance (EPR) was implemented by using 5,5‐dimethyl‐1‐pyrroline N‐oxide (DMPO) as a spin‐trapping agent. As depicted in Figure 4i, the presence of Fe‐MnPS_3_/PDA‐PEG nanosheets and H_2_O_2_ in a mildly acidic environment (pH = 5.0) results in a strong EPR signal and a typical 1:2:2:1 multiplet, which are characteristic features of ^•^OH. Notably, an even stronger EPR signal intensity is observed after NIR laser irradiation, further demonstrating the thermal‐augmented effect on accelerating the Fenton reaction for the production of ^•^OH. Besides, we have also conducted a comparative analysis of ^•^OH generation efficiency between pure MnPS_3_ and Fe‐MnPS_3_ nanosheets (as shown in Figure S17, Supporting Information). The findings demonstrate the nanosheets exhibit significantly improved ^•^OH generation capability after immobilization of Fe (II) ions. These results reveal that the remarkable Fenton catalytic capabilities exhibited by Fe‐MnPS_3_/PDA‐PEG nanosheets can be mainly attributed to the highly efficient Fenton reaction facilitated by the presence of Fe (II) ions on the surface of these nanosheets.

### 2.4 Cytotoxicity and In Vitro Inhibitory Effects of PTT/CDT on Cancer Cells

**Figure 5:**
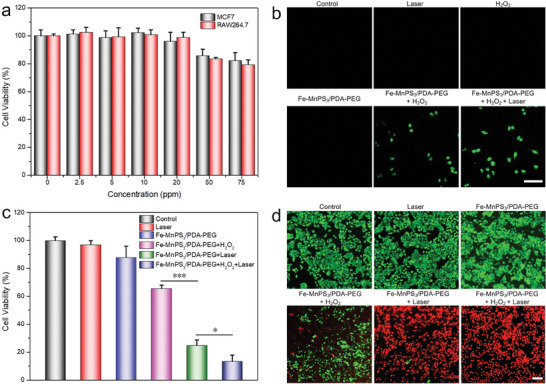
Cytotoxicity and in vitro PTT/CDT inhibition of cancer cells: a) Relative cell viability of MCF7 and RAW264.7 cells after incubation with Fe‐MnPS_3_/PDA‐PEG nanosheets at varying concentrations (0, 2.5, 5, 10, 20, 50, and 75 ppm). b) ROS fluorescence images of MCF7 cells after different treatments by using the DCFH‐DA probe, scale bar = 50 µm. c) Relative cell viabilities of MCF7 cells under different treatments (Control, Laser, Fe‐MnPS_3_/PDA‐PEG, Fe‐MnPS_3_/PDA‐PEG + H_2_O_2_, Fe‐MnPS_3_/PDA‐PEG + Laser, and Fe‐MnPS_3_/PDA‐PEG + H_2_O_2_ + Laser). (*n* = 5; *P < 0.05, ***P < 0.001) d) Corresponding fluorescence images of the cells stained with calcein‐AM (live cells, green fluorescence) and PI (dead cells, red fluorescence), scale bars = 50 µm).

Nanomaterials used in biomedicine should exhibit sufficient biocompatibility. Thus, the cytotoxicity of the Fe‐MnPS_3_/PDA‐PEG nanosheets was assessed on both normal and cancer cells. The Cell Counting Kit‐8 (CCK‐8) assay was employed to examine the relative viability of the MCF7 and RAW264.7 cells after being cultured with Fe‐MnPS_3_/PDA‐PEG nanosheets at various concentrations (0, 2.5, 5, 10, 20, 50, and 75 ppm) for 24 h. As shown in **Figure** **5**a, the cell viability remains largely unaffected by the presence of Fe‐MnPS_3_/PDA‐PEG nanosheets, with over 80% cell viability observed after 24 h incubation, thereby indicating low cytotoxicity of the nanosheets. To evaluate the cellular uptake of the nanoplatform, MCF7 cells were co‐incubated with the Fe‐MnPS_3_/PDA‐PEG nanosheets. As depicted in the bright‐field images in Figure S18a (Supporting Information), it is evident that the cancer cells can efficiently internalize the Fe‐MnPS_3_/PDA‐PEG nanosheets after incubation for 4 h. The uptake efficiency of Fe‐MnPS_3_/PDA‐PEG nanosheets was further confirmed by ICP‐MS, and the results illustrated in Figure S18b (Supporting Information) indicate the uptake of the nanosheets is dose‐dependent. Notably, when the concentration of the nanosheets reaches 40 ppm, the cellular uptake can be as high as 16.17 ± 3.8 pg/cell, revealing its excellent cellular uptake capability. To further confirm the presence of ^•^OH in the MCF7 cellular environment induced by Fe‐MnPS_3_/PDA‐PEG nanosheets and H_2_O_2_, the ROS fluorescence probe 2,7‐dichloro‐dihydro‐fluorescien diacetate (DCFH‐DA) was utilized to detect the ^•^OH generation. The DCFH‐DA probe can be readily oxidized by ROS, transforming into fluorescent 2,7‐dichlorofluorescein (DCF), emitting green fluorescence. As expected, the MCF7 cells appear green fluorescence after being co‐administered with Fe‐MnPS_3_/PDA‐PEG nanosheets and H_2_O_2_ for 4 h in Figure 5b and Figure S19 (Supporting Information), declaring the intracellular ROS production. Noteworthily, the green fluorescence is further enhanced upon 808 nm laser irradiation for 10 min, indicating that the temperature elevation induced by laser irradiation can efficiently enhance the generation of ^•^OH. It has been demonstrated that temperature increase can promote the catalytic activity of Fenton agents.^[^ ^34^ ^]^ Additionally, the temperature elevation results in enhanced release of Fe ions, thereby promoting the Fenton reaction. These results manifest the Fe‐MnPS_3_/PDA‐PEG nanosheets have great potential to be employed as a Fenton agent to initiate CDT by inducing intracellular ROS generation, which can be further augmented by the NIR‐triggered photothermal effect.

Motivated by the superior photothermal properties and chemodynamic performance of Fe‐MnPS_3_/PDA‐PEG nanosheets, their in vitro anticancer efficacy was further examined on MCF7 cancer cells. As presented in Figure 5c, a notable decline in cell viability is observed when co‐incubating the Fe‐MnPS_3_/PDA‐PEG nanosheets with either H_2_O_2_ or laser alone. Notably, a more substantial decrease in cell viability is found in the groups where Fe‐MnPS_3_/PDA‐PEG nanosheets are co‐incubated with both H_2_O_2_ and laser, revealing a synergistic enhancement in cancer cell eradication. To further assess the impact of Fe (II) ions immobilization and PDA modification on cell cytotoxicity, we conducted a series of experiments, including MnPS_3_ + H_2_O_2_ + Laser, Fe‐MnPS_3_‐PEG + H_2_O_2_ + Laser, and MnPS_3_/PDA‐PEG + H_2_O_2_ + Laser. The results shown in Figure S20 (Supporting Information) reveal that pure MnPS_3_ nanosheets still maintain a high cell survival rate when exposed to laser irradiation and H_2_O_2_. In contrast, the Fe‐MnPS_3_‐PEG and MnPS_3_/PDA‐PEG nanosheets exhibit significant cytotoxicity toward the cells under the same conditions. These findings provide compelling evidence that Fe (II) ions immobilization and PDA modification can significantly enhance cell cytotoxicity. Besides, to confirm the therapeutic effect, cells were co‐stained with Calcine AM and propidium iodide (PI) to distinguish dead (red) and living (green) cells (Figure 5d). Severe cell death is observed (almost no green color) when cells are simultaneously incubated with Fe‐MnPS_3_/PDA‐PEG nanosheets and H_2_O_2_ along with 808 nm laser irradiation. In contrast, no substantial cell death is observed (almost no red color) in the other three groups, including the Control group, Laser group, and Fe‐MnPS_3_/PDA‐PEG group. These results are consistent with the CCK8 results, certifying the superior synergistic photothermal‐chemodynamic effect of Fe‐MnPS_3_/PDA‐PEG nanosheets.

### 2.5 Fluorescence Imaging and Magnetic Resonance Imaging

**Figure 6:**
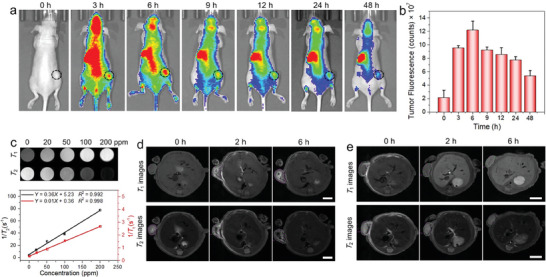
In vivo bioimaging: a) Fluorescence images of MCF7 tumor‐bearing nude mice administrated with Cy5.5‐labeled Fe‐MnPS_3_/PDA‐PEG nanosheets at various time intervals. b) Quantification of fluorescence intensity in tumor areas after the injection of Fe‐MnPS_3_/PDA‐PEG nanosheets using ROI. c) *T*_1_‐ and *T*_2_‐weighted MRI images and corresponding relaxation rates of Fe‐MnPS_3_/PDA‐PEG nanosheets at different concentrations (0, 20, 50, 100, 200 ppm). d,e) In vivo *T*_1_‐ and *T*_2_‐weighted MR images of the tumor‐bearing mouse before and after d) intratumor and e) intravenous administration of Fe‐MnPS_3_/PDA‐PEG nanosheets (the dotted circles indicate tumor sites). Scale bar = 4 mm.

The incorporation of multiple treatment modalities and the clinical requirement for visual diagnosis have become increasingly essential in the development of “all‐in‐one” nanoplatforms. Herein, to further investigate the potential of Fe‐MnPS_3_/PDA‐PEG nanosheets for in vivo imaging, the MCF7 xenograft tumor model was established by planting the MCF7 cells into the right leg of nude mice. To visualize the in vivo distribution of Fe‐MnPS_3_/PDA‐PEG nanosheets, Cy5.5, a widely used NIR fluorescent dye, was employed for labeling. As shown in Figure S21 (Supporting Information), the Fe‐MnPS_3_/PDA‐PEG nanosheets labeled with Cy5.5 exhibit strong fluorescence, while no obvious fluorescence is observed from the pure Fe‐MnPS_3_/PDA‐PEG nanosheets and water, which indicates the successful labeling of Cy5.5 on the nanosheets. After intravenous injection of Cy5.5‐labeled Fe‐MnPS_3_/PDA‐PEG into MCF7 tumor‐bearing mice via the tail vein (12.5 mg kg^−1^), fluorescent images were acquired at different time points by IVIS fluorescence imaging (FLI). As shown in **Figure** **6**a, at 3 h post‐injection, a weak fluorescence signal is detected at the tumor site, indicating gradual accumulation of the Fe‐MnPS_3_/PDA‐PEG nanosheets due to the permeability and retention (EPR) effect. It has been reported that nanomaterials within the size range of 60–400 nm extravasate and accumulate in tumors via the EPR effect,^[^ ^35^ ^]^ allowing for the accumulation of Fe‐MnPS_3_/PDA‐PEG nanosheets in the tumor areas. Meanwhile, the fluorescence intensity progressively increases over time, and the maximum fluorescence is obtained after 6 h post‐injection, signifying efficient enrichment of the nanoplatform in the tumor region and optimal timing for PTT. In contrast, free Cy5.5 cannot accumulate efficiently in the tumor area owing to its short blood circulation time (Figure S22, Supporting Information).^[^ ^36^ ^]^ Subsequently, as time passes, the fluorescence intensity in the tumor gradually diminishes, with only weak fluorescence observed after 48 h, suggesting that Fe‐MnPS_3_/PDA‐PEG nanosheets can be metabolized gradually over time. The ROI (region of interest) was further employed for semi‐quantitative analysis of the tumor areas. Figure 6b illustrates similar variations in fluorescence intensity, which is in accordance with the fluorescent images.

Magnetic resonance imaging (MRI), a crucial tool for early tumor diagnosis, provides exceptional soft‐tissue morphological details and functional information on lesions with high spatial resolution and unlimited penetration depth, making it widely utilized in clinical diagnostics.^[^ ^37^ ^]^ To unveil the potential of Fe‐MnPS_3_/PDA‐PEG nanosheets as a contrast agent for MRI, the dual mode *T*_1_‐ and *T*_2_‐weighted MR images, along with the corresponding longitudinal and transverse relaxation rates at various concentrations were evaluated using a clinical 7.0 T MRI scanner. As revealed in Figure 6c, both *T*_1_‐ and *T*_2_‐ weighted MR images exhibit evident concentration‐dependent signals of brightness and darkness, respectively. The corresponding curve fitting of relaxation time versus concentration demonstrates a positive linear correlation, revealing the highly desirable contrast‐enhanced capability of Fe‐MnPS_3_/PDA‐PEG for both *T*_1_ and *T*_2_‐weighted imaging. Subsequently, the *T*_1_ and *T*_2_‐weighted contrast‐enhanced effects of the Fe‐MnPS_3_/PDA‐PEG nanosheets were investigated in vivo. The MCF7 tumor‐bearing Balb/c nude mice were administered with Fe‐MnPS_3_/PDA‐PEG nanosheets via intratumoral and intravenous routes, respectively. As shown in Figure 6d, the *T*_1_‐weighted images exhibit enhanced brightness at the tumor sites after 2 h post‐intratumoral injection, with further intensification at 6 h. Simultaneously, the *T*_2_‐weighted images show darkened regions at the tumor sites with increased contrast over time. The MRI signal enhancement in the tumor region was further semi‐quantified by assessing the average pixel brightness value, as illustrated in Figure S23a (Supporting Information). In *T*_1_ images, the increased average pixel brightness value over time indicates an elevated MRI signal intensity, whereas the decreased average pixel brightness value signifies an increased MRI signal intensity in *T*_2_ images. Compared to the intratumoral administration, the intravenous administration of Fe‐MnPS_3_/PDA‐PEG nanosheets leads to relatively weaker but more homogeneous signal distribution within the entire tumor in both *T*_1_‐ and *T*_2_‐weighted images (Figure 6e; Figure S23b, Supporting Information). This observation suggests that intravenous injection efficiently facilitates the widespread accumulation of the nanosheets within the entire tumor region. Collectively, these results unequivocally demonstrate the superior FLI/MRI capabilities of Fe‐MnPS_3_/PDA‐PEG nanosheets, underscoring their substantial potential for advancing tumor diagnosis.

### 2.6 Tumor Therapy using Fe‐MnPS_3_/PDA‐PEG Nanosheets

**Figure 7:**
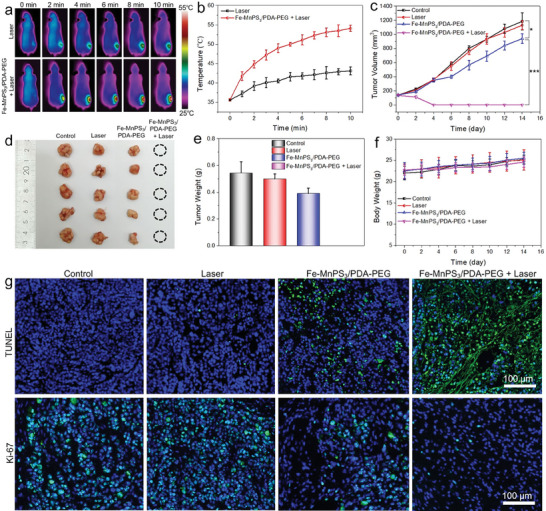
In vivo photothermal/chemodynamic synergistic therapy: a) Infrared thermal images and b) time‐dependent photothermal heating curves in MCF7 tumor‐bearing nude mice under 808 nm laser irradiation (1.0 W cm^−2^, 10 min) after 6 h post‐injection of PBS and Fe‐MnPS_3_/PDA‐PEG nanosheets. c) Tumor growth profiles after different treatments (*n* = 5). Results are shown as mean ± SD. Statistical significance was calculated using Student's two‐tailed t‐test (*p < 0.05, ***p < 0.001). d) Photographs of tumors dissected from different groups at the end of the experiments (*n* = 5). e) Tumor weights collected from different groups. f) Body weight changes of the mice from different groups during the treatments (*n* = 5). g) TUNEL and Ki‐67 staining of tumor sites at 24 h post‐treatment.

Encouraged by the outstanding in vitro antitumor efficacy of Fe‐MnPS_3_/PDA‐PEG nanosheets, we proceeded to assess their in vivo therapeutic potential. MCF7 tumor‐bearing mice were randomly assigned into four groups (*n* = 5 per group) and treated as follows: a) Control, b) Laser, c) Fe‐MnPS_3_/PDA‐PEG nanosheets, and d) Fe‐MnPS_3_/PDA‐PEG + Laser. The tumor‐bearing mice were intravenously injected with Fe‐MnPS_3_/PDA‐PEG nanosheets (12.5 mg kg^−1^) or PBS solution prior to NIR laser irradiation. After 6 h post‐injection, a time point corresponding to the maximal accumulation and retention of Fe‐MnPS_3_/PDA‐PEG nanosheets in the tumor (from Figure 6), the mice were intraperitoneally anesthetized, and their tumor sites were exposed to an 808 nm laser (1.0 W cm^−2^) for 10 min. To evaluate the in vivo photothermal performance, an infrared thermal imaging camera was utilized to capture the real‐time thermal images and monitor temperature variation at the tumor sites. As depicted in **Figure** **7**a,b, the tumor‐site temperature of mice in Laser group exhibits only a slight temperature elevation, whereas the tumor‐site temperature in the Fe‐MnPS_3_/PDA‐PEG + Laser group shows a remarkable elevation to ≈54 °C, revealing the highly efficient photothermal effect within the tumors. After the treatments, the tumor sizes were recorded every two days using a digital caliper to assess the tumor growth. Compared to the Control group and Laser group, the Fe‐MnPS_3_/PDA‐PEG group exhibits significant inhibition of tumor growth through Fenton reaction‐based CDT. More importantly, the tumors in the Fe‐MnPS_3_/PDA‐PEG + Laser group were completely eliminated upon additional NIR laser irradiation, highlighting the synergistic effects of both PTT and CDT (Figure 7c; Figure S24, Supporting Information). The photographs of dissected tumors (Figure 7d) and corresponding tumor weights analysis (Figure 7e) after the various treatments

further confirm the excellent therapeutic efficacy of synergistic PTT/CDT. To further evaluate the impact of Fe (II) ions immobilization and PDA modification on tumor ablation, some control groups experiments were conducted, including MnPS_3_ + Laser, Fe‐MnPS_3_‐PEG + Laser, and MnPS_3_/PDA‐PEG + Laser. The results depicted in Figure S25 (Supporting Information) demonstrate that pure MnPS_3_ nanosheets exhibit rapid tumor growth when exposed to laser irradiation. However, the Fe‐MnPS_3_‐PEG and MnPS_3_/PDA‐PEG nanosheets show significant inhibition of tumor growth under the same conditions. These findings reveal that Fe (II) ions immobilization and PDAmodification can significantly enhance the therapeutic efficacy for tumor elimination. Additionally, we monitored the mice's weight changes every two days to assess their overall health status during the treatment period. As shown in Figure 7f, none of the mice exhibits abnormal weight loss throughout the therapeutic period, suggesting minimal side effects.

To further explore the underlying therapeutic mechanisms of the synergistic PTT/CDT, tumor sections dissected from different groups of mice were collected at 24 h post‐treatment. H&E staining reveals that the Fe‐MnPS_3_/PDA‐PEG + Laser group exhibits more severe tumor necrosis and morphological changes than the control groups (Figure S26, Supporting Information). Additionally, immunofluorescence staining was further conducted by adopting terminal deoxynucleotidyl transferase biotin‐dUTP nick end labeling (TUNEL) assay and Ki‐67 antibody to evaluate the mechanisms mediating tumor suppression. TUNEL staining was employed to reveal the cell apoptosis in tumor tissues, while Ki‐67 staining was applied to divulge the cellular proliferation in tumor sections. As disclosed in Figure 7g, the Fe‐MnPS_3_/PDA‐PEG group exhibits notable cell apoptosis, primarily attributed to the CDT effect, while a larger amount of cell apoptosis is readily observed in Fe‐MnPS_3_/PDA‐PEG + Laser group ascribing to the combined therapeutic effect of PTT and CDT. Conversely, no significant cell apoptosis was observed in the Control group and Laser group. Additionally, from the Ki‐67 images, only a few Ki‐67‐positive cells are observed in the Fe‐MnPS_3_/PDA‐PEG + Laser group, unlike the other groups, indicating a potent inhibitory effect on cell proliferation. All of these results manifest the remarkable synergistic effects by combining PTT with CDT with the aid of Fe‐MnPS_3_/PDA‐PEG nanosheets as both Fenton agent and photothermal agent.

### 2.7 In Vivo Biosafety, Biodistribution, and Clearance Assessment

**Figure 8:**
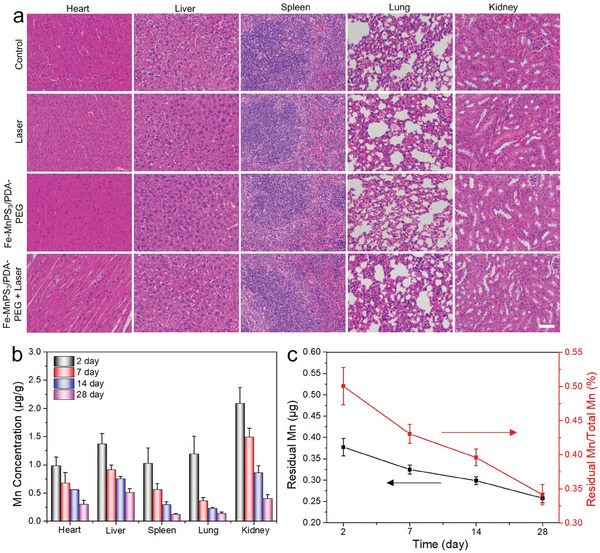
Assessment of in vivo biosafety, biodistribution, and clearance: a) Representative H&E stained images of major organs, including the heart, liver, spleen, lung, and kidney of the mice in different groups after treatment for 14 days. Scale bar = 50 µm. b) Mn concentrations in the main organs at different time points of 2, 7, 14, and 28 days after intravenous injection of Fe‐MnPS_3_/PDA‐PEG nanosheets (12.5 mg kg^−1^). c) The residual amounts of Mn and corresponding residual ratios plotted versus time.

The biosafety of nanomaterials is highly critical for their potential use in biomedicine, thus the in vivo toxicity was further evaluated. At the end of the various treatments, the mice were euthanized and the main organs, including the heart, liver, spleen, lung, and kidney were collected for H&E staining. As illustrated in **Figure** **8**a, the control and treated groups do not show any appreciable organ damage or inflammatory lesions, indicating the good biocompatibility of Fe‐MnPS_3_/PDA‐PEG nanosheets in vivo. To further verify the biological safety of the Fe‐MnPS_3_/PDA‐PEG nanosheets, we conducted blood biochemistry and blood routine assessments. The blood biochemical parameters, including alanine transaminase (ALT), aspartate transaminase (AST), blood urea nitrogen (BUN), and creatinine (CREA), are evaluated (Figure S27a, Supporting Information). Besides, the blood routine parameters such as white blood cells (WBC), red blood cells (RBC), haemoglobin (HGB), haematocrit (HCT), mean corpuscular volume (MCV), mean corpuscular haemoglobin (MCH), mean corpuscular haemoglobin concentration (MCHC), and platelets (PLT), were measured (Figure S27b, Supporting Information). Compared to the control group, almost all the results obtained from the Fe‐MnPS_3_/PDA‐PEG nanosheets groups are normal with no significant difference, indicating that the Fe‐MnPS_3_/PDA‐PEG nanosheets do not produce obvious deleterious effects.

Besides, nanomaterials that cannot be efficiently metabolized or cleared from the body will induce potential health hazards owing to excessive accumulation, limiting their clinical applications. Therefore, the biodistribution and clearance behavior of the Fe‐MnPS_3_/PDA‐PEG nanosheets were further investigated to assess their long‐term safety in biomedicine. Fe‐MnPS_3_/PDA‐PEG nanosheets were initially administered intravenously to healthy mice with a final dose of 12.5 mg kg^−1^. The mice were then sacrificed after 2, 7, 14, and 28 days, with their major organs harvested for biodistribution analysis. The liver and kidney are the primary organs of accumulation. After 2 days post‐injection, both the liver and kidney already show relatively low Mn concentrations, with 1.4 µg g^−1^ in the liver and 2.1 µg g^−1^ in the kidney, respectively (Figure 8b). Subsequently, the Mn concentrations in the main organs gradually decrease over time, suggesting the efficient clearance of Fe‐MnPS_3_/PDA‐PEG nanosheets from the body. By the 28th day, the Mn concentrations in the liver and kidney have dropped to only 0.5 µg g^−1^ in the liver and 0.4 µg g^−1^ in the kidney, respectively, implying that the majority of Fe‐MnPS_3_/PDA‐PEG nanosheets have been excreted from the body. To survey the detailed in vivo clearance efficiency, the time‐dependent residual amount of initial injected Fe‐MnPS_3_/PDA‐PEG nanosheets in mice was evaluated (Figure 8c). The residual amounts of Mn in mice were determined by summing the Mn amount in all major organs from Figure 8b. After 28 days post‐administration, the residual ratio of Mn is decreased to only 0.34%, verifying that the Fe‐MnPS_3_/PDA‐PEG nanosheets are metabolizable and exhibit significantly higher clearance efficiency compared to many other 2D nanomaterials.^[^ ^38^ ^]^

## 3 Conclusion

In summary, we have successfully developed an MnPS_3_‐based multifunctional nanoplatform for efficient multimodal imaging‐guided cancer synergistic PTT/CDT. MnPS_3_ nanosheets were synthesized through liquid exfoliation and immobilized with Fe (II) with high efficacy via electrostatic absorption. After surface modification with PDA and PEG, the Fe‐MnPS_3_/PDA‐PEG nanosheets exhibited extraordinarily high photothermal conversion efficiency and pH/NIR‐responsive Fenton catalytic activity. In vitro experiments indicated that Fe‐MnPS_3_/PDA‐PEG nanosheets exhibited negligible cytotoxicity and remarkable PTT/CDT efficacy against cancer cells. Upon systemic administration in vivo, highly efficient tumor eradication without recurrence was accomplished due to its potent photothermal effect and high Fenton catalytic efficiency, along with their synergistic effect. Moreover, Fe‐MnPS_3_/PDA‐PEG nanosheets demonstrated their potential as a versatile multifunctional theranostic agent, enabling efficient MRI, FLI, and PTI‐guided tumor therapy. In vivo experiments revealed that Fe‐MnPS_3_/PDA‐PEG nanosheets possess good biocompatibility without inducing noticeable hepatic and renal dysfunctions or tissue injury. More importantly, this nanoplatform composed of biocompatible elements (Mn, P, and S) can be naturally excreted from the body to avoid long‐term retention and potential toxicity. This work opens up new horizons for the application of MnPS_3_‐based multifunctional nanoplatform, offering significant promise in the field of cancer theranostics with potential for clinical applications.

## 4 Experimental Section

### Materials

The bulk MnPS_3_ crystals (99.9999%) were purchased from Shenzhen SixCarbon Technology Co., Ltd (Shenzhen, China). The ferrous sulfate heptahydrate (FeSO_4_^•^7H_2_O), dopamine hydrochloride (DA‐HCl, >98%), PEG‐NH_2_ (99%), N‐methyl‐2‐pyrrolidone (NMP, 99.5%, anhydrous), 3,3′,5,5′‐Tetramethylbenzidine (TMB), methylene blue (MB), and hydrogen peroxide (H_2_O_2_) were acquired from Aladdin Reagent Co., Ltd. (Shanghai, China). The phosphate‐buffered saline (PBS, pH 7.4), fetal bovine serum (FBS), DMEM, trypsin‐EDTA, and penicillin‐streptomycin were obtained from Gibco Life Technologies (AG, Switzerland). Calcein‐AM and PI were bought from Invitrogen (USA). The 2′, 7′‐dichlorofluorescein diacetate (DCFH‐DA) was purchased from Med Chem Express (USA). The Cy5.5 fluorescence dye was purchased from Shaanxi Xinyan Bomei Biotechnology Co., Ltd (China). All the chemicals used in this study were analytical reagent grade and used without further purification.

### Characterization

Scanning electron microscopy (SEM) images were acquired from a ZEISS Merlin. Transmission electron microscopy (TEM) and energy‐dispersive X‐ray spectroscopy (EDS) images were obtained by an FEI Talos. X‐ray diffraction (XRD) spectra were performed by Rigaku Smartlab. Raman spectra were conducted on a HORIBA LabRAM HR Evolution. The XPS spectra were measured by using PHI 5000 Versaprobe III. The zeta potential was determined by a BI‐200SM Goniometer. The atomic force microscopy (AFM) images were taken by MFP‐3D Stand Alone system in tapping mode. Fourier transform infrared spectroscopy (FTIR) was performed on MDTC‐EQ‐M13‐01 (Thermo Scientific). The absorption spectra were obtained from a UV–vis–NIR spectrophotometer (PerkinElmer LAMBDA 750s, USA). The element concentrations were determined by an inductively coupled plasma mass spectrometry (ICP‐MS) (7700X, PerkinElmer). The temperature changes and thermal images were recorded on the infrared thermal imaging instrument (Fotric 220).

### Preparation of MnPS_3_ Nanosheets

Seventy milligrams of the MnPS_3_ crystals were placed in a mortar, ground for 20 min, and transferred to a glass vial containing 70 mL of NMP to reach a final concentration of 1 mg mL^−1^. The dispersion was sonicated in an ice bath at 300 W for 2 h and then sonicated with a sonic tip for 8 h with a power of 300 W. The supernatant containing the MnPS_3_ nanosheets was collected by centrifugation at 6000 rpm min^−1^ for 10 min, and the precipitate was re‐collected by centrifugation for another 15 min at 11 000 rpm min^−1^.

### Preparation of Fe‐MnPS_3_ Nanosheets

Ten milligrams of MnPS_3_ nanosheets were dispersed in 6 mL of ultrapure water, followed by the addition of 6 mL of 1 mg mL^−1^ FeSO_4_^•^7H_2_O solution. The solution was then magnetically stirred for 15 min. Subsequently, the mixture was centrifuged at 9000 rpm min^−1^ for 8 min to collect the precipitate, which was then washed twice with water.

### Preparation of Fe‐MnPS_3_/PDA Nanosheets

For dopamine modification, 10 mg of Fe‐MnPS_3_ nanosheets were dispersed in an 8 mL dopamine solution (0.3 mg mL^−1^, pH 8.5, 10 mm Tris‐HCl). After 30 min of sonication, the resulting precipitate was collected by centrifugation at 9000 rpm min^−1^ for 8 min and subsequently purified with water.

### Preparation of Fe‐MnPS_3_/PDA‐PEG Nanosheets

Four milliliters of PEG‐NH_2_ solution (10 mg mL^−1^) was mixed with 10 mg of Fe‐MnPS_3_/PDA nanosheets and stirred magnetically for 20 min. The mixture was centrifuged several times to remove the excess PEG molecules.

### Photothermal Properties of Fe‐MnPS_3_/PDA‐PEG Nanosheets

The Fe‐MnPS_3_/PDA‐PEG nanosheets dispersed in ultrapure water at varying concentrations (0, 12.5, 25, 50, and 100 ppm) were exposed to an 808 nm laser with a power density of 1.0 W cm^−2^. To investigate the influence of power density on the photothermal properties, the Fe‐MnPS_3_/PDA‐PEG nanosheets aqueous solutions were irradiated with an 808 nm laser at different power densities (0.0, 0.5, 1.0, 1. 5, and 2.0 W cm^−2^). During the irradiation process, the laser spot was adjusted to cover the entire sample surface for uniform heating, and the real‐time temperature variations were recorded using an infrared thermal imaging camera.

### Chemodynamic Properties of Fe‐MnPS_3_/PDA‐PEG Nanosheets

To further quantitatively analyze the production of ^•^OH, a conventional colorimetric method based on the oxidation of TMB was conducted. Typically, the Fe‐MnPS_3_/PDA‐PEG (500 ppm), H_2_O_2_ (1 mm), and TMB (20 mm) were efficiently mixed, and the total volume was 1 mL. After the reaction in darkness, the photographs and UV–vis–NIR absorbance spectra of oxidized TMB were acquired, respectively. The ^•^OH generation was further assessed via a common MB degradation method. Specifically, Fe‐MnPS_3_/PDA‐PEG nanosheets (500 ppm) were mixed with the MB (10 ppm) and H_2_O_2_ (1 mm) solutions and kept in darkness. The ^•^OH‐induced MB degradation was determined by monitoring the decrease in the absorption peak of MB using a UV–vis–NIR spectrophotometer. Besides, electron paramagnetic resonance (EPR) was then applied to further verify the generation of·^•^OH. 25 µL 5,5‐dimethyl‐1‐pyrroline N‐oxide (DMPO) solution (200 mm) was mixed with 25 µL solutions including H_2_O_2_ (1 mm) and Fe‐MnPS_3_/PDA‐PEG nanosheets (500 ppm) under varying conditions. Subsequently, the mixture was instantly added to a capillary tube, and the EPR spectrum was acquired by a Bruker EMXplus‐10/12 spectrometer.

### Cytotoxicity Assay

The MCF7 and RAW264.7 cells were obtained from the China‐type culture collection (CTCC) and the cytotoxicity of the Fe‐MnPS_3_/PDA‐PEG nanosheets was evaluated through a standard CCK‐8 assay. Briefly, cells were seeded into 96‐well plates with a density of 1 × 10^4^ cells per well and cultured for 24 h in a humid chamber of 5% CO_2_ at 37 °C. Subsequently, the original culture media were replaced with 200 µL of fresh media containing different concentrations of Fe‐MnPS_3_/PDA‐PEG nanosheets (0, 2.5, 5, 10, 20, 50, and 75 ppm). After incubation for 24 h, the media were substituted by the 10% CCK‐8 medium and incubated for another 60 min. Cell viability was measured by the absorbance at 450 nm using a microplate reader (FilterMax F5, Molecular Devices, USA).

### Cellular Uptake of Fe‐MnPS_3_/PDA‐PEG Nanosheets

The MCF7 cells were cultured on a 48‐well plate with a density of 1 × 10^5^ cells per well overnight. Afterward, the original culture media were replaced with varying concentrations of Fe‐MnPS_3_/PDA‐PEG nanosheets (2.5, 5, 10, 20, and 40 ppm) and incubated for another 4 h. Subsequently, the cells were washed with PBS solution for at least three times, and the bright field images were captured by an Olympus IX71 microscope. Following this, the cells were trypsinized and re‐suspended in 3 mL of HNO_3_ (65%) overnight to solubilize the intracellular Fe‐MnPS_3_/PDA‐PEG nanosheets. The quantitative analysis of the nanosheets within the cells was further performed by ICP‐MS.

### In Vitro ROS Generation Mediated by Fe‐MnPS_3_/PDA‐PEG Nanosheets

MCF7 cells were seeded into 96‐well plates with a density of 1 × 10^4^ cells per well and incubated to adhere. Subsequently, the cells were incubated with DCFH‐DA (10 µm) for 30 min in darkness. After that, the cells were subjected to different treatments including PBS, Laser irradiation (1.0 W cm^−2^, 10 min), H_2_O_2_ (100 µm), Fe‐MnPS_3_/PDA‐PEG nanosheets (50 ppm), Fe‐MnPS_3_/PDA‐PEG + Laser irradiation (1.0 W cm^−2^, 10 min), and Fe‐MnPS_3_/PDA‐PEG + H_2_O_2_ + Laser irradiation (1.0 W cm^−2^, 10 min). Then, the culture media were removed, and the cells were rinsed with PBS for subsequent fluorescence observation.

### In Vitro Inhibition of Cancer Cells

To assess synergistic PTT/CDT efficacy, MCF7 cells (1 × 10^4^ cells per well) were incubated overnight. Afterward, the initial culture media were removed, and the cells were exposed to different treatments, including PBS, Laser (1.0 W cm^−2^, 10 min), Fe‐MnPS_3_/PDA‐PEG nanosheets (50 ppm), Fe‐MnPS_3_/PDA‐PEG + H_2_O_2_, Fe‐MnPS_3_/PDA‐PEG + Laser, and Fe‐MnPS_3_/PDA‐PEG + H_2_O_2_ + Laser. The cells were incubated for another 24 h and cell viability was quantitatively analyzed using the CCK‐8 assay. Additionally, the cells were co‐stained with Calcein AM and PI for 30 min, rinsed twice with PBS, and visualized by inverted fluorescence microscopy (AxioObserver7, Zeiss, Germany).

### Tumor Model

Female Balb/c nude mice (5‐6 weeks old) were purchased from Beijing Weitong Lihua Laboratory Animal Technology Co., Ltd. (Beijing, China) and all the animal experiments were performed in accordance with guidelines evaluated and approved by the Animal Care and Use Committee of the Southern University of Science and Technology (IACUC NO. SUSTech‐JY202105004). MCF7 cells (1 × 10^7^ cells in 100 µL PBS) were subcutaneously injected into the right leg of each female Balb/c nude mouse to induce tumor formation.

### In Vivo FLI

Fluorescent‐labeled Fe‐MnPS_3_/PDA‐PEG nanosheets were prepared by adding 0.1 mg mL^−1^ of Cy5.5 to the solution, followed by magnetic stirring for 3 h in darkness. Excess dye molecules were discarded by centrifugation and washed repeatedly with water. The Cy5.5‐labeled Fe‐MnPS_3_/PDA‐PEG nanosheets were re‐suspended in PBS and intravenously administered to the MCF7 tumor‐bearing mice at a final dose of 12.5 mg kg^−1^. At different time points (0, 3, 6, 9, 12, 24, and 48 h post‐injection), fluorescence images were captured through an IVIS Spectrum (PerkinElmer, USA), and fluorescence intensity was quantified using Living Image 4.2.

### In Vitro and In Vivo MRI Performance

The MRI characteristics and relaxation times of Fe‐MnPS_3_/PDA‐PEG nanosheets at varying concentrations (0, 20, 50, 100, and 200 ppm) were assessed using an MRI system. For in vivo MRI, the MCF7 tumor‐bearing mice were anesthetized by 2% isoflurane in an oxygen environment and placed in the small animal coil in the supine position. After 0, 2, and 6 h intratumoral (2 mg kg^−1^) or intravenous (10 mg kg^−1^) injection of Fe‐MnPS_3_/PDA‐PEG nanosheets, the *T*_1_‐ and *T*_2_‐ MRI images of tumor areas were acquired by a 7.0 T MRI system. The imaging parameters were set as follows: for *T*_1_ images, TR = 400 ms, TE = 8 ms, slice thickness = 1.0 mm, FOV = 20 × 20; for *T*_2_ images, TR = 4000 ms, TE = 35 ms, slice thickness = 1.0 mm, FOV = 20 × 20.

### In Vivo Tumor Therapy

Once the tumors reached a size of ≈150 mm^3^, the mice were assigned randomly to four groups (*n* = 5 per group): a) Control, b) Laser, c) Fe‐MnPS_3_/PDA‐PEG nanosheets, and d) Fe‐MnPS_3_/PDA‐PEG + Laser. The MCF7 tumor‐bearing mice were intravenously injected with Fe‐MnPS_3_/PDA‐PEG nanosheets (12.5 mg kg^−1^) or PBS solution prior to NIR laser irradiation. After 6 h post‐injection, the mice were intraperitoneal anesthetized and the tumor sites were irradiated with the 808 nm laser (1.0 W cm^−2^) for 10 min. The real‐time thermal images and temperature measurements of the tumor sites were recorded by an infrared thermal imaging camera. The changes in body weight were recorded every two days to assess the health status of the mice. The tumor size was measured by a digital caliper during the therapeutic period, and the tumor volume was calculated using the formula: tumor volume (*V*) = (tumor length) × (tumor width)^2^/2. After that, the histopathological analysis was executed by TUNEL and Ki‐67.

### In Vivo Biosafety Evaluation

After 14 days of the administration, all the mice were euthanized and the major organs containing heart, liver, spleen, lung, and kidney were collected for hematoxylin and eosin (H&E) staining. These organs were first fixed in a 4% paraformaldehyde solution, embedded in paraffin, and then sliced, stained with hematoxylin and eosin (H&E), and examined using a fluorescence microscope equipped with a digital camera.

### In Vivo Biodistribution and Clearance Study

Twenty healthy mice were intravenously injected with Fe‐MnPS_3_/PDA‐PEG nanosheets (12.5 mg kg^−1^) via the tail vein. At 2, 7, 14, and 28 days post‐administration, the mice were sacrificed, and the main organs, including the heart, liver, spleen, lung, and kidney were dissected, weighed, and solubilized in HNO_3_ (65%) overnight. The tissue lysates were dried, dissolved with HNO_3_ (1%), and filtered with a 0.22 µm filter membrane to remove any undigested tissue. The concentrations of Mn in these organs were quantitatively determined by ICP‐MS.

### Statistical Analysis

Sample sizes (*n* = 5) were selected based on guidance from the established practices in the literature. Statistical analyses were conducted employing OriginPro 9.0 Software. Quantitative data were shown as means ± standard deviation (SD) and the statistical significance was calculated by using Student's two‐tailed t‐test. In all statistical assessments, *p < 0.05 was considered statistically significant, **p < 0.01 was considered highly significant, and ***p < 0.001 was denoted very highly significant.

## Conflict of Interest

The authors declare no conflict of interest.

## Supporting information

Supporting Information

## Data Availability

The data that support the findings of this study are available from the corresponding author upon reasonable request.
