# Supplementary material for: Polydopamine‐Modified 2D Iron (II) Immobilized MnPS3 Nanosheets for Multimodal Imaging‐Guided Cancer Synergistic Photothermal‐Chemodynamic Therapy
Source: Adv Sci (Weinh). 2023 Dec 11;11(7):2306494. doi: 10.1002/advs.202306494 (PMC10870060; doi:10.1002/advs.202306494)
Supplement: Supplementary file 1 — Supporting Information [file ADVS-11-2306494-s001.pdf]

ADVANCED SCIENCE
Open Access

Supporting Information

for *Adv. Sci.*, DOI 10.1002/advs.202306494

Polydopamine-Modified 2D Iron (II) Immobilized $MnPS_3$ Nanosheets for Multimodal Imaging-Guided Cancer Synergistic Photothermal-Chemodynamic Therapy

*Hanhan Xie, Ming Yang, Xiaoli He, Zhen Zhan, Huaide Jiang, Yanmei Ma and Chengzhi Hu**

Polydopamine-Modified Two-Dimensional Iron (II) Immobilized $MnPS_3$ Nanosheets for Multimodal Imaging-Guided Cancer Synergistic Photothermal-Chemodynamic Therapy

Hanhan Xie, Ming Yang, Xiaoli He, Zhen Zhan, Huaide Jiang, Yanmei Ma, and Chengzhi Hu.*

Prof. H. Xie, Dr. M. Yang, Dr. X. He, Dr. Z. Zhan, Dr. H. Jiang, Dr. Y. Ma, Prof. C. Hu
Shenzhen Key Laboratory of Biomimetic Robotics and Intelligent Systems, Department of Mechanical and Energy Engineering, Southern University of Science and Technology, Shenzhen 518055, China
Prof. H. Xie, Dr. M. Yang, Dr. X. He, Dr. Z. Zhan, Dr. H. Jiang, Dr. Y. Ma, Prof. C. Hu
Guangdong Provincial Key Laboratory of Human-Augmentation and Rehabilitation Robotics in Universities, Southern University of Science and Technology, Shenzhen 518055, China
E-mail: hucz@sustech.edu.cn

Calculation of the photothermal conversion efficiency (η):

Following Roper's report,[1] we calculate the photothermal conversion efficiency (η) of Fe-$MnPS_3$/PDA-PEG nanosheets by using the following equation (1):

$$\eta = (hS(T_{max}-T_{surr})-Q_{dis})/I(1-10^{-A}) \quad (1)$$

where h is the heat transfer coefficient, S is the surface area of the container. T_{max} is the equilibrium temperature, T_{surr} is the surrounding ambient temperature, and Q_{dis} represents the heat dissipated by the solvent measured independently using a quartz cuvette.[2] I is the laser power density, and A is the absorbance of Fe-$MnPS_3$/PDA-PEG nanosheets at 808 nm. In equation (1), only the hS remains unknown. The value of hS can be derived following equation (2) ~ equation (4):

$$\theta = (T - T_{surr})/(T_{max} - T_{surr}) \quad (2)$$

$$\tau_s = - t/\ln\theta \quad (3)$$

$$hS = \sum mC_p/\tau_s \quad (4)$$

where θ represents the dimensionless driving force temperature, and T is the instant temperature during the cooling time (t). τ_s is the sample system time constant, which is determined to be 274 s. m and C_p are the mass (1 g) and heat capacity (4.2 J g^{-1}) of the aqueous solution, respectively. Thus, hS can be calculated to be 15.3 mW/°C. Then, the photothermal conversion efficiency (η) of Fe-$MnPS_3$/PDA-PEG nanosheets is calculated to be 40.7%.

Reference

[1] D. K. Roper, W. Ahn, M. Hoepfner, *J. Phys. Chem. C.* **2007**, *111*, 3636.

[2] B. Geng, W. Shen, P. Li, F. Fang, H. Qin, X. K. Li, D. Pan, L. Shen, *ACS Appl. Mater. Interfaces* **2019**, *11*, 44949.

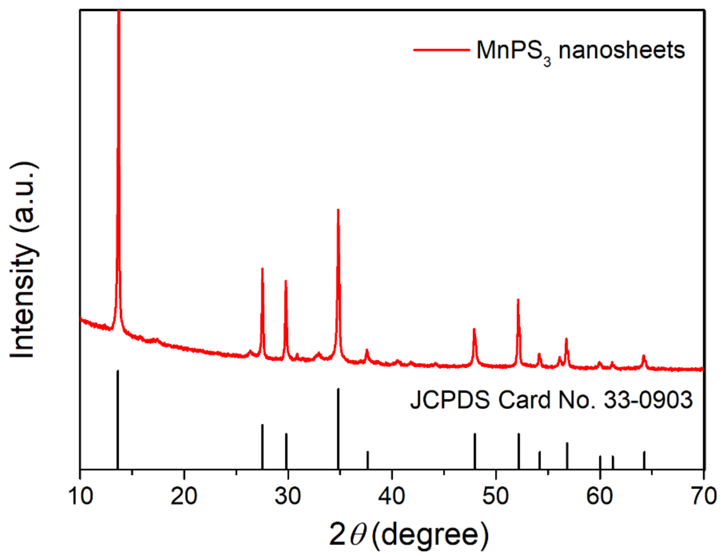

Figure S1. XRD pattern of $MnPS_3$ nanosheets.

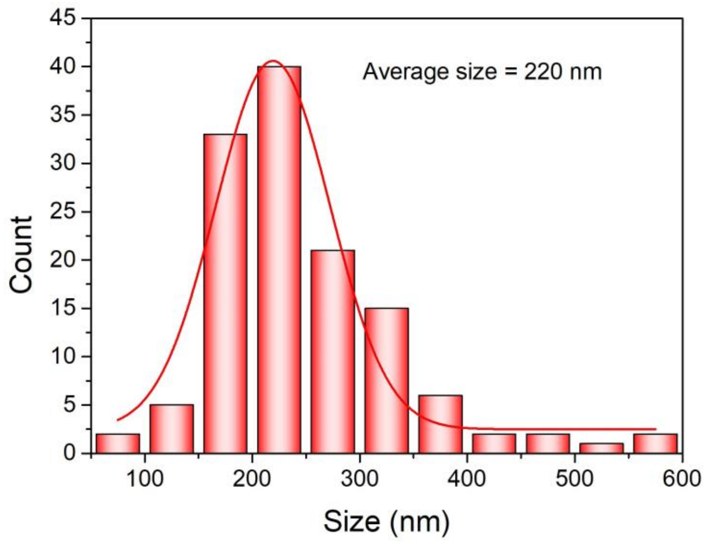

Figure S2. Statistical analysis of the lateral size of $MnPS_3$ nanosheets obtained from the SEM images.

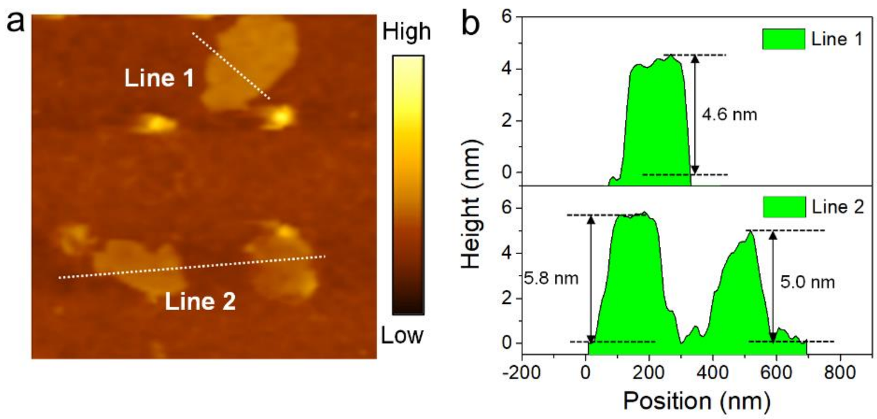

Figure S3. a) AFM image of Fe-$MnPS_3$ nanosheets. b) Height profiles of Fe-$MnPS_3$ nanosheets along the white lines in a).

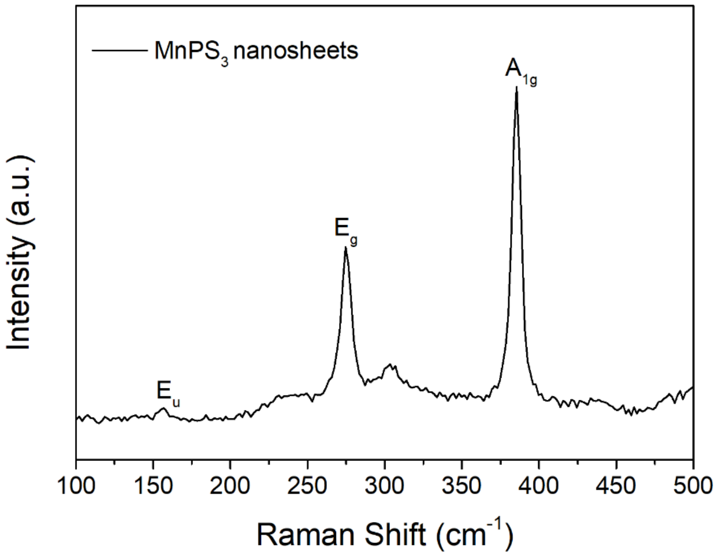

Figure S4. Raman spectrum of $MnPS_3$ nanosheets.

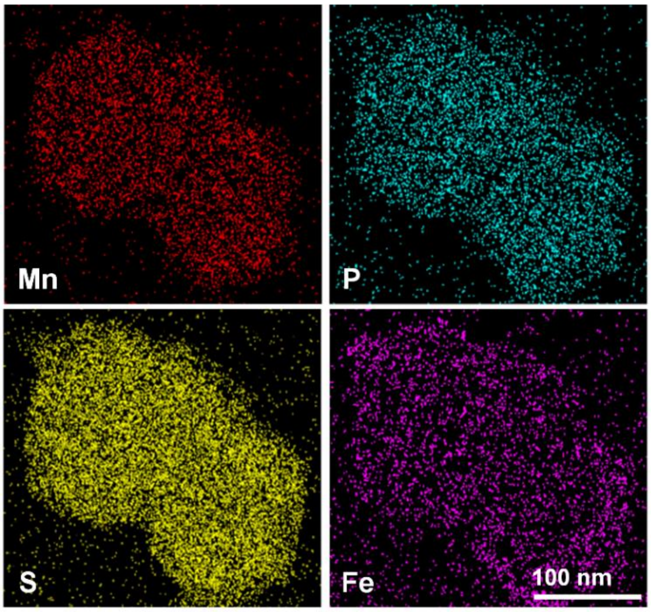

Figure S5. EDS mapping of Mn, P, S, and Fe for Fe-$MnPS_3$ nanosheets, respectively.

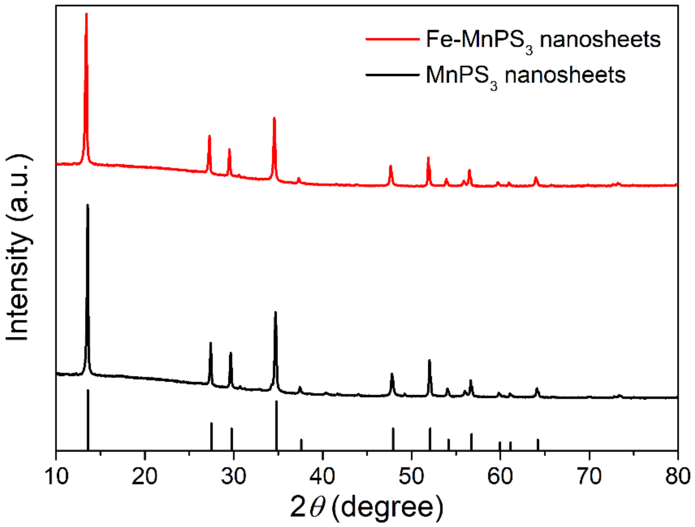

Figure S6. XRD patterns of $MnPS_3$ nanosheets and Fe-$MnPS_3$ nanosheets.

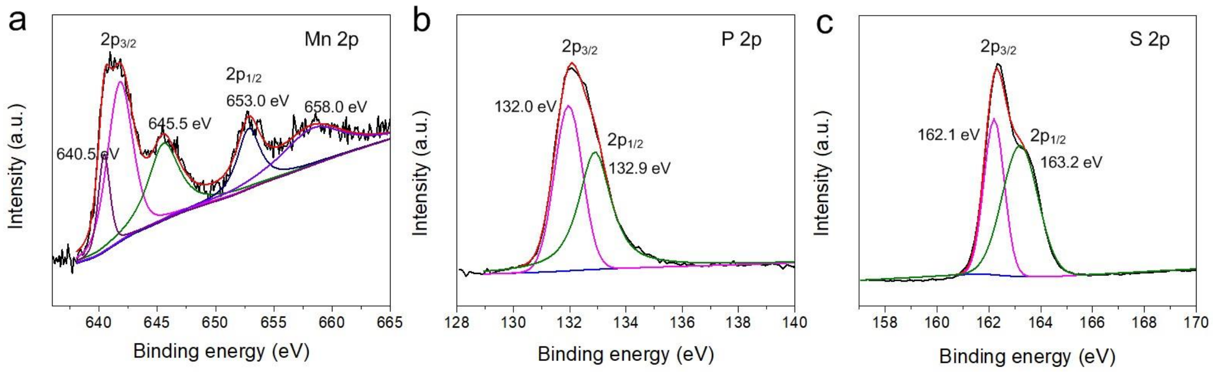

Figure S7. High-resolution XPS spectra of a) Mn 2p, b) P 2p, and c) S 2p in the Fe-$MnPS_3$ nanosheets.

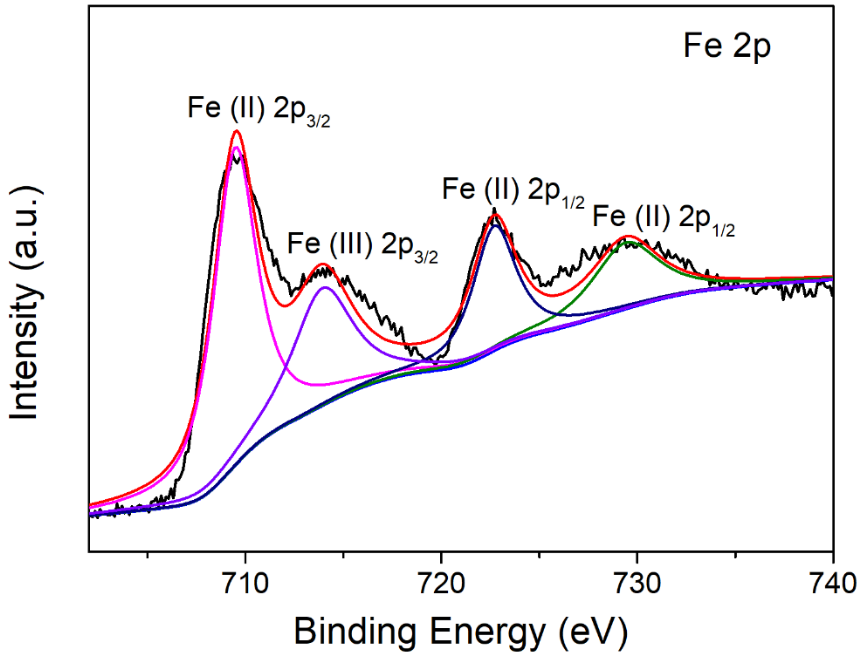

Figure S8. High-resolution XPS spectrum of Fe 2p in the Fe-$MnPS_3$ nanosheets.

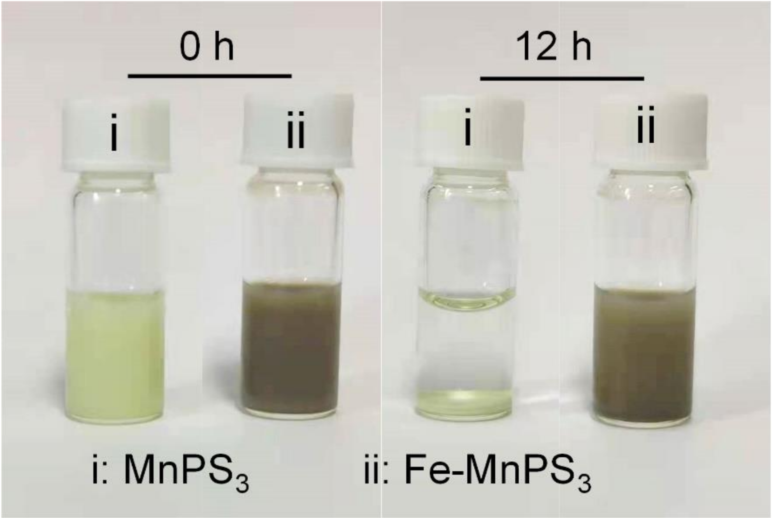

Figure S9. Photographs of $MnPS_3$ (i) and Fe-$MnPS_3$ (ii) dispersed in water for 0 h and 12 h.

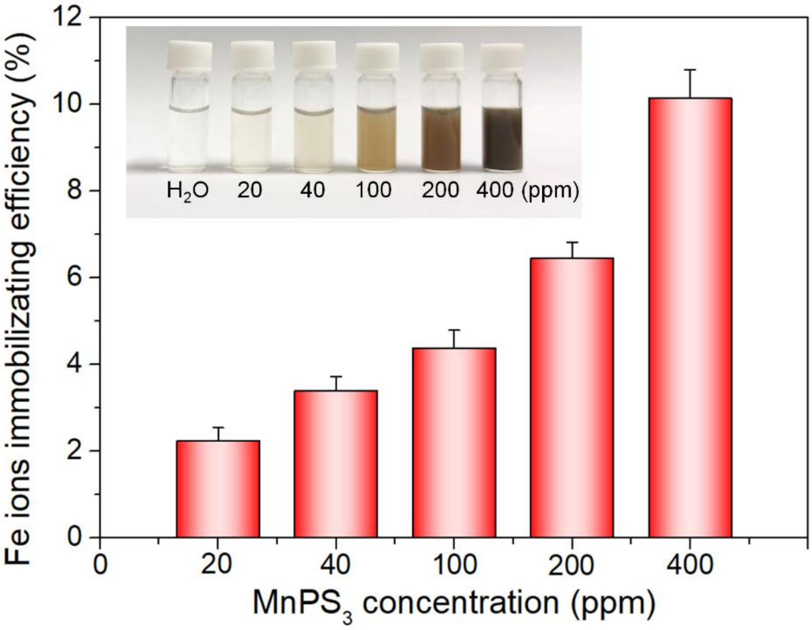

Figure S10. Fe ions immobilizing efficiency of $MnPS_3$ nanosheets dispersed in water at varied concentrations (0, 20, 40, 100, 200, and 400 ppm). Inset: Photographs of Fe-$MnPS_3$ nanosheets with different concentrations.

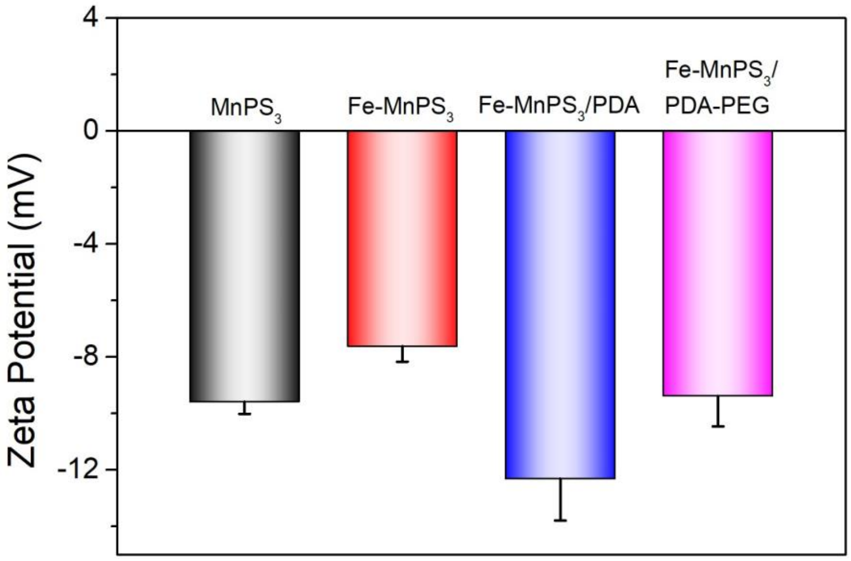

Figure S11. Zeta potentials of $MnPS_3$, Fe-$MnPS_3$, Fe-$MnPS_3$/PDA, and Fe-$MnPS_3$/PDA-PEG nanosheets.

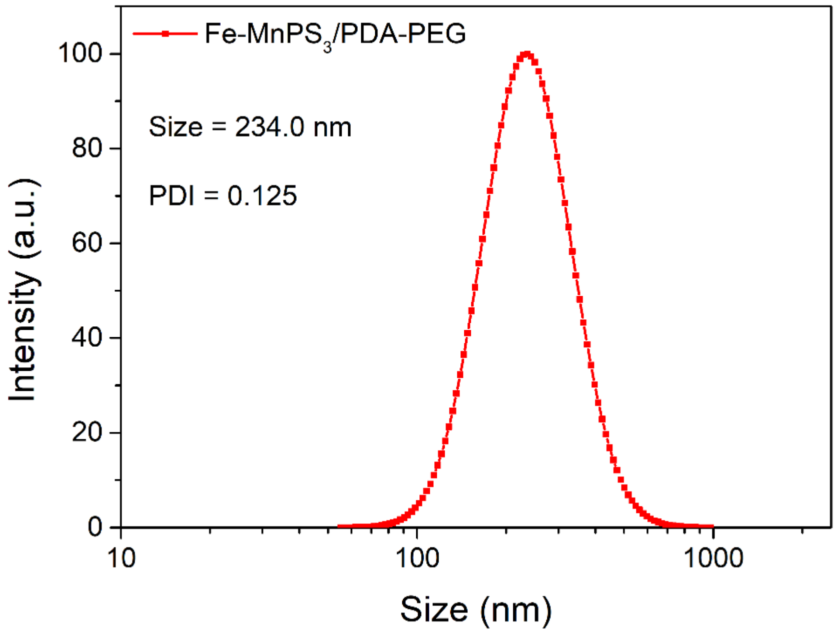

Figure S12. Particle-size distribution of Fe-$MnPS_3$/PDA-PEG nanosheets in aqueous solution.

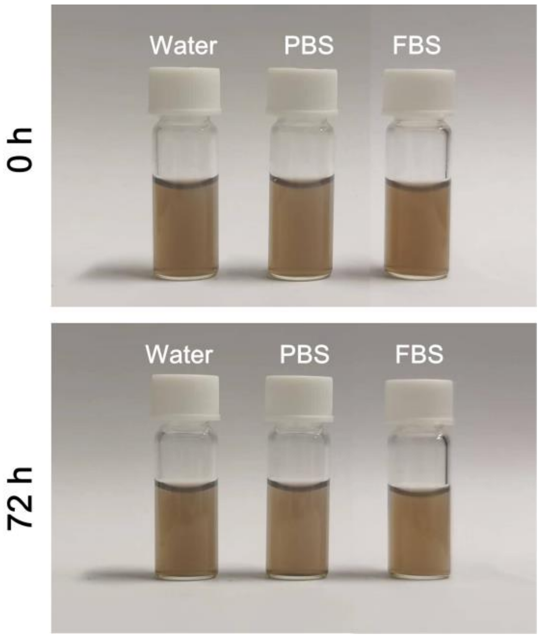

Figure S13. Photographs of the Fe-MnPS$_3$/PDA-PEG nanosheets dispersed in different solvents for 0 h and 72 h.

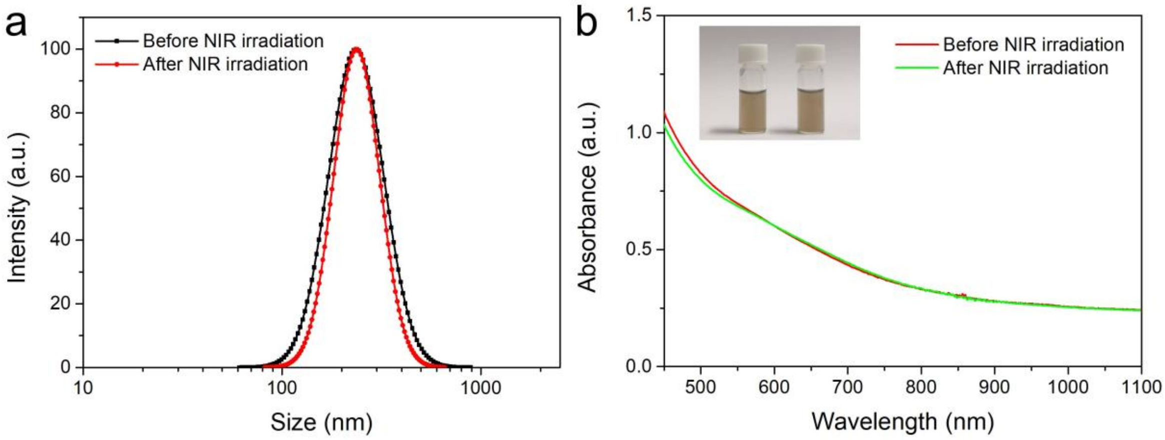

Figure S14. a) Size changes of Fe-MnPS$_3$/PDA-PEG nanosheets before and after NIR irradiation. b) Absorbance spectra of Fe-MnPS$_3$/PDA-PEG nanosheets before and after NIR irradiation. Inset: the corresponding photographs.

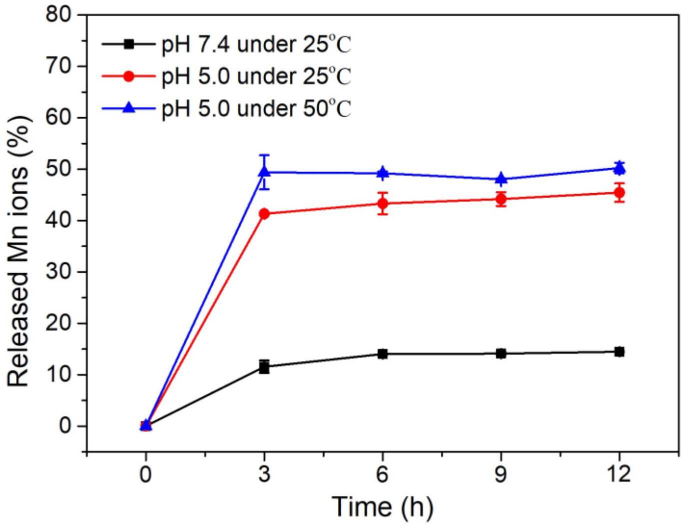

Figure S15. Quantified Mn ions released from Fe-$MnPS_3$/PDA-PEG nanosheets under different pH and temperature conditions.

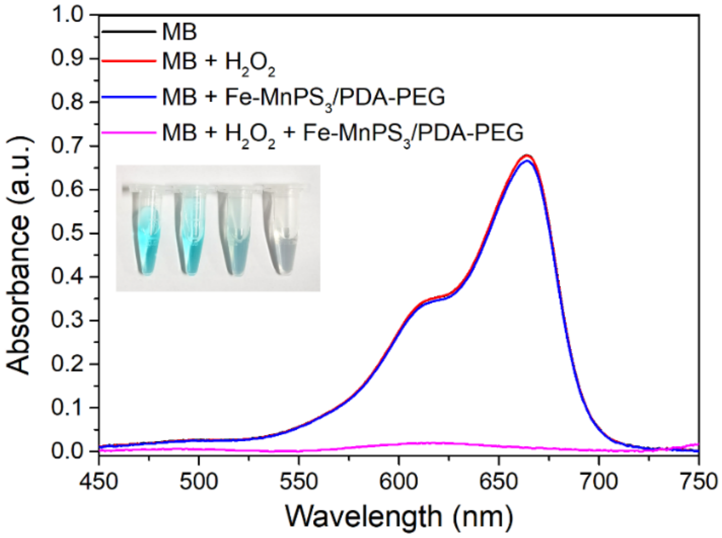

Figure S16. UV-vis absorption spectra of MB under different conditions (only MB, MB + H_2O_2, MB + Fe-$MnPS_3$/PDA-PEG, and MB + Fe-$MnPS_3$/PDA-PEG + H_2O_2). Inset: corresponding color changes of samples.

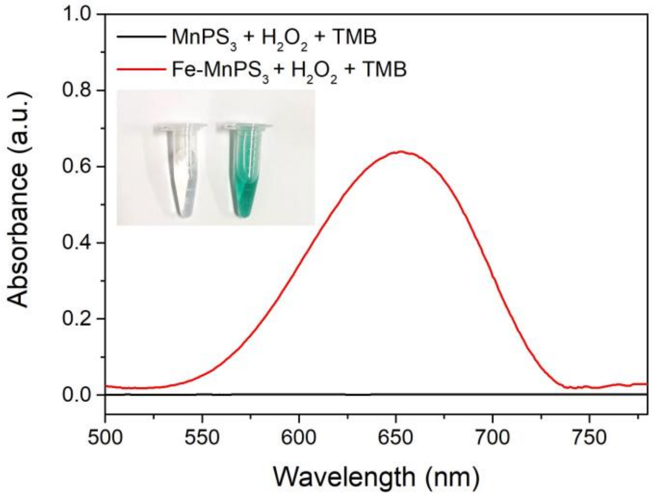

Figure S17. UV-vis absorption spectra of oxTMB with two groups ($MnPS_3 + H_2O_2$ + TMB and Fe-$MnPS_3 + H_2O_2$ + TMB). Inset: corresponding photographs of the two groups.

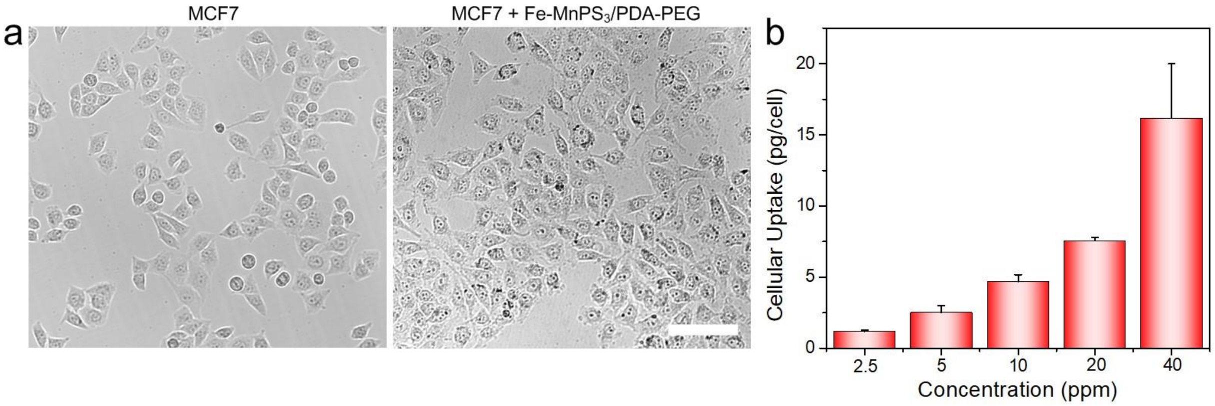

Figure S18. a) Bright-field images of MCF7 cells before and after uptake of Fe-$MnPS_3$/PDA-PEG nanosheets. b) Quantitative determination of MCF7 cell uptake of Fe-$MnPS_3$/PDA-PEG nanosheets at different concentrations. The scale bar is 100 μm for all images.

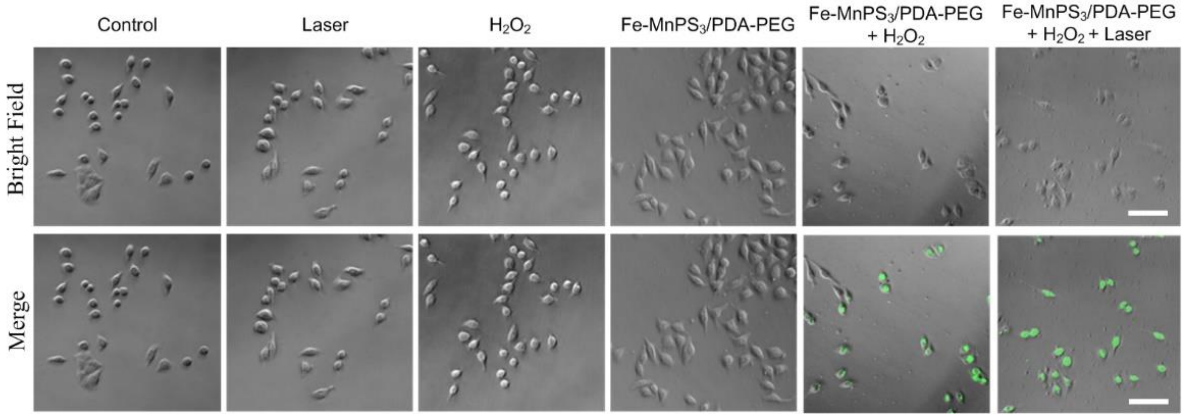

Figure S19. Bright-field and their merged images of MCF7 cells after different treatments. DCFH-DA is employed to detect the generation of hydroxyl radicals (•OH) through the observation of green fluorescence. Scale bar = 50 μm.

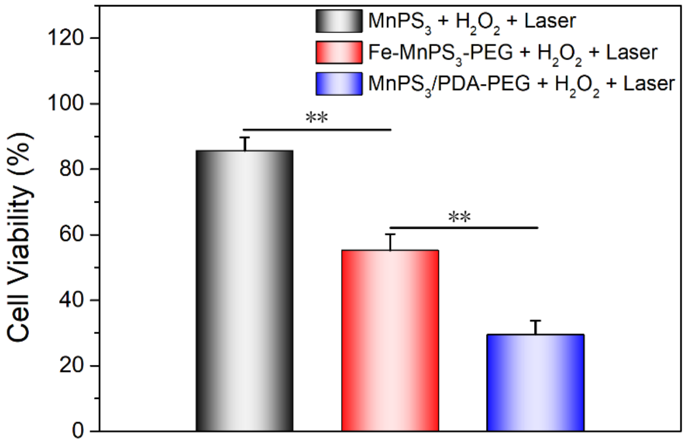

Figure S20. Relative cell viabilities of MCF7 cells under different treatments ($MnPS_3 + H_2O_2$ + Laser, Fe-$MnPS_3$-PEG + H_2O_2 + Laser, and $MnPS_3$/PDA-PEG + H_2O_2 + Laser. ($n = 5$; **$P < 0.01$)

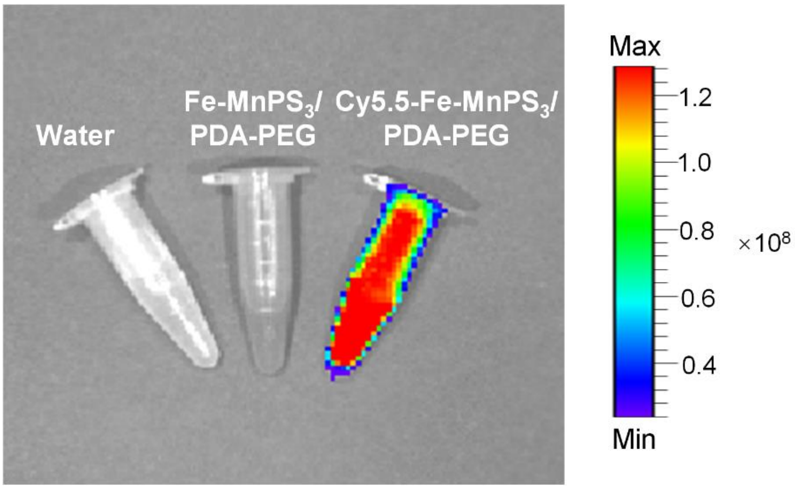

Figure S21. Fluorescence images of water, Fe-$MnPS_3$/PDA-PEG nanosheets, and Cy5.5-Fe-$MnPS_3$/PDA-PEG nanosheets.

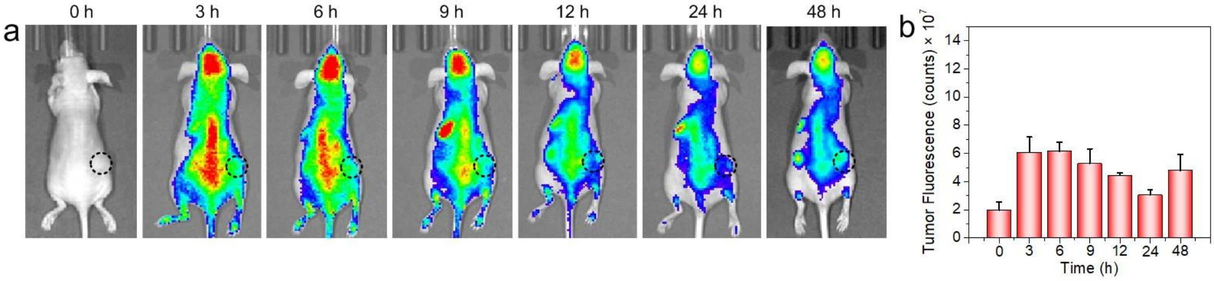

Figure S22. a) Fluorescence images of MCF7 tumor-bearing nude mice administrated with free Cy5.5 at different time intervals. b) Quantification of fluorescence intensity in tumor areas after the injection of free Cy5.5 by using ROI.

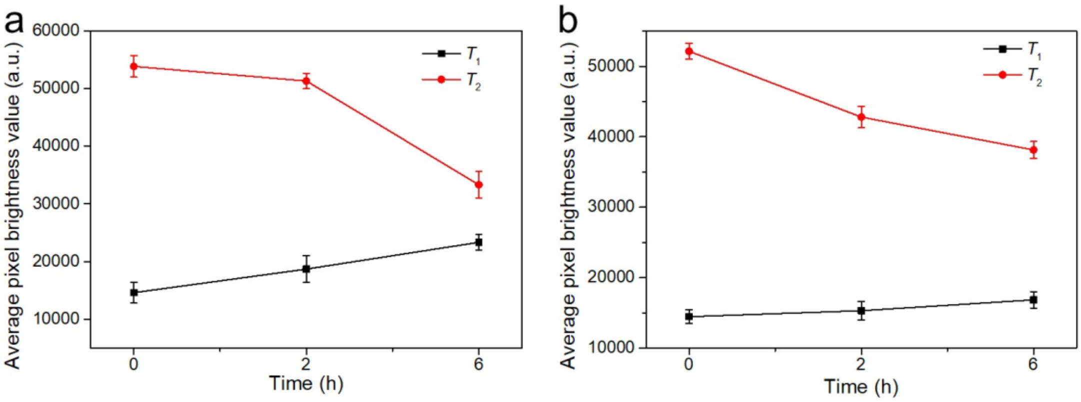

Figure S23. Semi-quantitative analysis of the MRI-signal intensity in tumor areas by assessing the average pixel brightness value after (a) intratumoral or (b) intravenous injection of Fe-$MnPS_3$/PDA-PEG nanosheets at 0, 2, and 6 h, respectively.

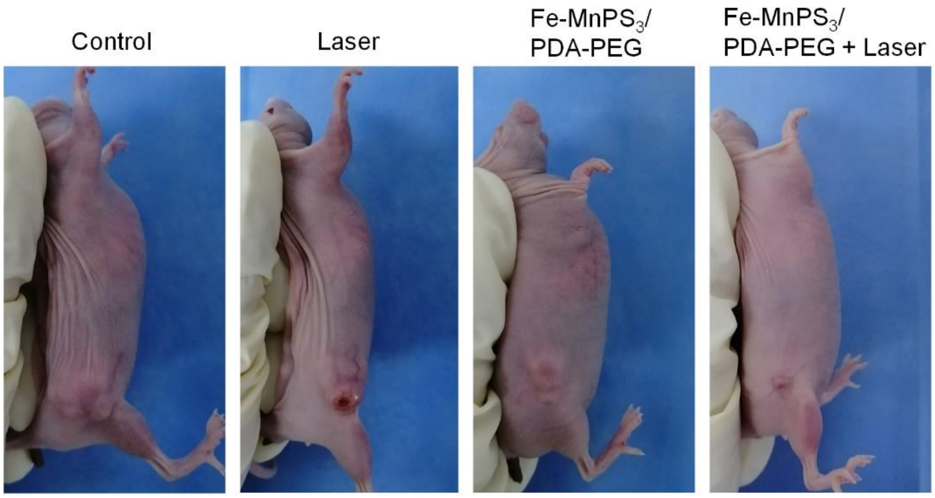

Figure S24. Photographs of MCF7 tumor-bearing mice and tumor regions after different treatments for 14 days.

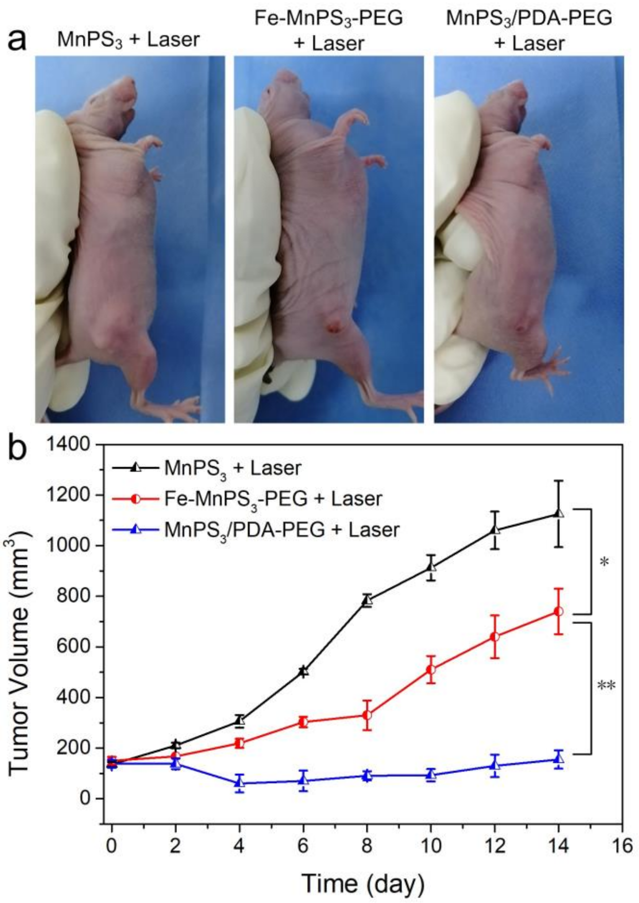

Figure S25. a) Typical photographs of MCF7 tumor-bearing mice after different treatments for 14 days. b) Time-dependent tumor growth curves in different groups of mice.

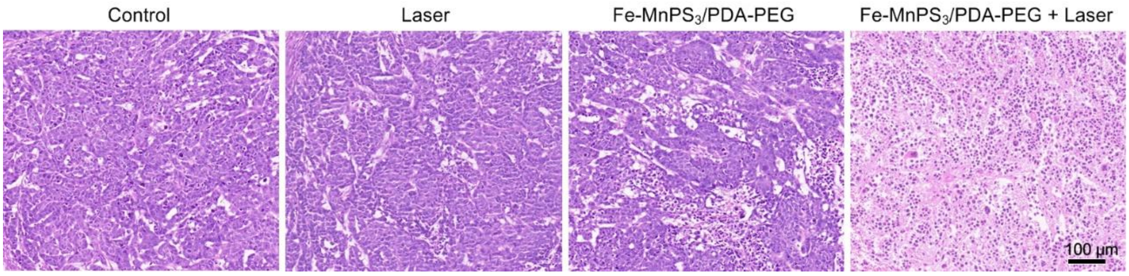

Figure S26. H&E staining of MCF7 tumor regions after different treatments in 24 h.

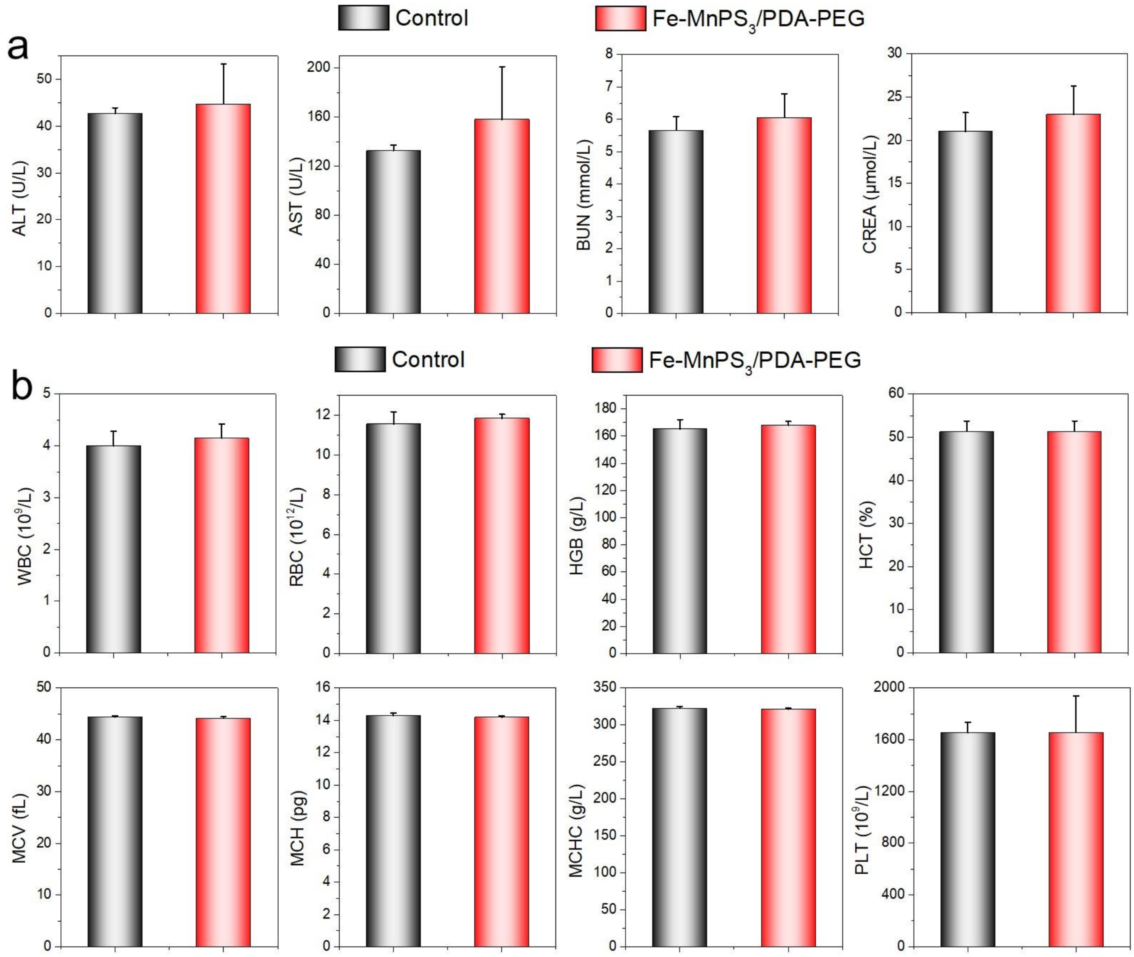

Figure S27. a) Blood biochemistry data of the mice after intravenous injection of Fe-$MnPS_3$/PDA-PEG nanosheets for 14 days with parameters including ALT, AST, BUN, and CREA. b) Blood routine analysis of the mice after intravenous injection of Fe-$MnPS_3$/PDA-PEG nanosheets for 14 days. The following parameters were measured: WBC, RBC, HGB, HCT, MCV, MCH, MCHC, and PLT.

Table S1. The contents of Fe and Mn in the Fe-$MnPS_3$/PDA-PEG nanosheets.

Element	Sample 1	Sample 2	Sample 3	Average value
Fe (%)	30.0%	30.1%	30.1%	30.1%
Mn (%)	30.2%	30.3%	30.2%	30.2%
